# Empirical Comparison of Exposure Set Definitions in the Prevalent New‐User Design

**DOI:** 10.1002/pds.70339

**Published:** 2026-02-27

**Authors:** John Tazare, Daniel C. Gibbons, Liam Smeeth, M. Sanni Ali, Iain A. Gillespie, Marianne Cunnington, John Logie, Elizabeth J. Williamson, Ian J. Douglas

**Affiliations:** ^1^ Faculty of Epidemiology and Population Health London School of Hygiene and Tropical Medicine London UK; ^2^ Real‐World Data Measurement & Analytics, GSK London UK; ^3^ GSK London UK; ^4^ Analysis Group Inc. London UK

**Keywords:** electronic health records, exposure sets, new‐user designs, prevalent new‐user design

## Abstract

**Purpose:**

Prevalent new‐user (PNU) designs aim to provide a wider assessment of treatment effects by incorporating users of a newer study drug who previously received the comparator. Similarity in terms of prior use of the comparator is accounted for via exposure sets based on time in study, prior prescription number, or a hybrid incorporating both plus calendar time. Given a current lack of consensus, we examine choice of exposure set definition using a study investigating upper gastrointestinal bleeding (UGIB) risk between users of non‐steroidal anti‐inflammatory drugs (NSAIDs) and cyclooxygenase‐2 inhibitors (COX‐2is).

**Methods:**

We identified a cohort of individuals with osteoarthritis initiating NSAIDs or COX‐2is between 2000 and 2004 from the UK Clinical Practice Research Datalink. Considering prescription‐based, time‐based, and hybrid exposure set definitions, we estimated time‐conditional propensity scores (TCPS) using conditional logistic regression and matched COX‐2i users 1:1 to NSAID users. Analyses estimated the hazard ratio (HR) of UGIB bleed comparing COX‐2i and NSAID users overall, in the incident new‐user and prevalent new‐user subgroups.

**Results:**

We identified 100 185 individuals who received a prescription for either COX‐2is or NSAIDs; 25 742 patients were incident new‐users of COX‐2is and 17 952 were PNUs. Prescription‐ and time‐based exposure sets resulted in the highest proportion of COX‐2i users successfully matched (86% vs. 76% using hybrid definition). We observed variability in the point estimates obtained under the different exposure set definitions; however, conclusions remained consistent.

**Conclusions:**

Given the potential for differences in the matched cohorts and substantive results obtained under different exposure set definitions, we encourage increased use of sensitivity analyses in PNU studies to explore the robustness of results to this decision.

## Key Points

- This study investigates the impact of exposure set definition in the prevalent new‐user (PNU) design, considering prescription‐based, time‐based, and hybrid definitions in a study using UK electronic health record (EHR) data.
- In our case study, we observed some variability in the point estimates obtained under the different exposure set definitions, highlighting the need for increased adoption of sensitivity analyses to explore the robustness of results to this decision.
- Given the dynamic prescribing practice of settings where PNU designs are commonly applied, we highlight the importance of accounting for calendar time in exposure set definitions. In our case study, the best balance for calendar time was achieved under the hybrid exposure set definition.
- We provide R code for implementing the PNU design via GitHub, which addresses a key barrier to wider application and development of the design.

## Plain Language Summary

This study looks at how different ways of defining exposure sets in the prevalent new‐user (PNU) study design can affect study findings. Using UK electronic health record data, we compared three approaches: prescription‐based, time‐based, and a hybrid method that combines both. We found that the choice of method led to some differences in results, suggesting it is important to explore how sensitive findings are to this decision. Because prescribing patterns can change over time, we also emphasise the need to consider calendar time when defining exposure sets. To help other researchers use the PNU design more easily, we have provided software for implementation.

## 1 Purpose

The prevalent new‐user (PNU) design extends the population of inference of the active comparator new‐user (ACNU) design by including initiators of a novel treatment of interest who were previously on an older comparator treatment, that is, switchers [1, 2, 3]. Development of the design was motivated, in part, by the need to address research questions relating to a new treatment, where application of an ACNU design can suffer few eligible participants and insufficient power to answer the question of interest.

**FIGURE 1:**
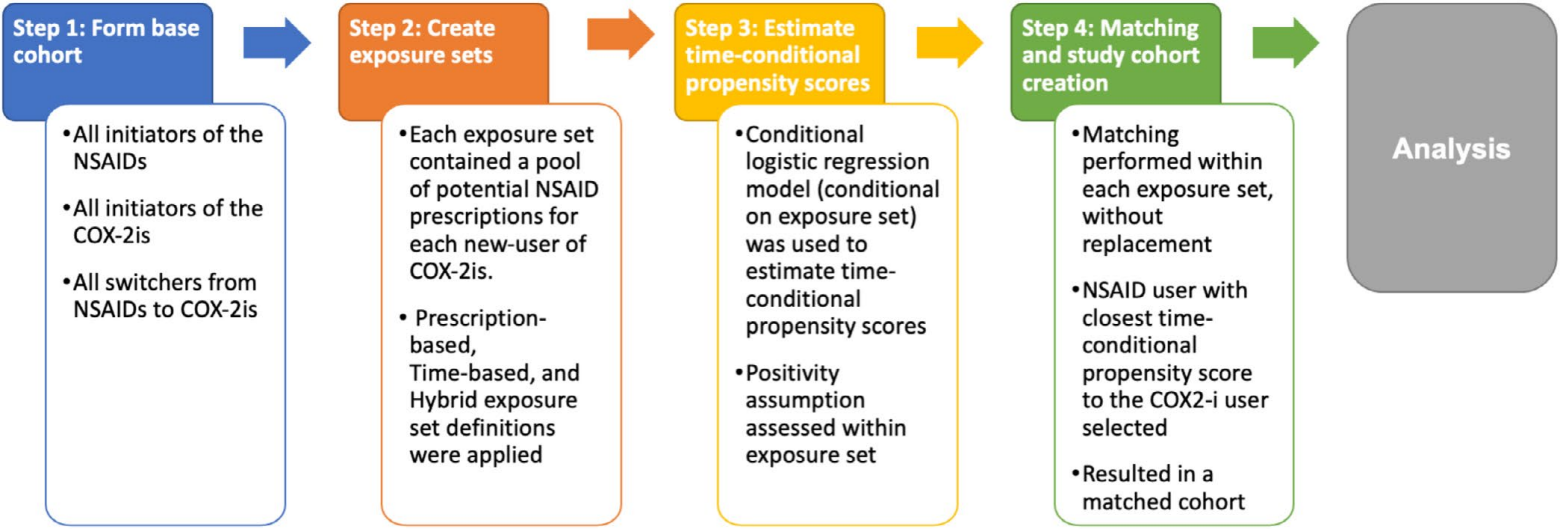
Summary of prevalent new‐user design implementation steps.

**FIGURE 2:**
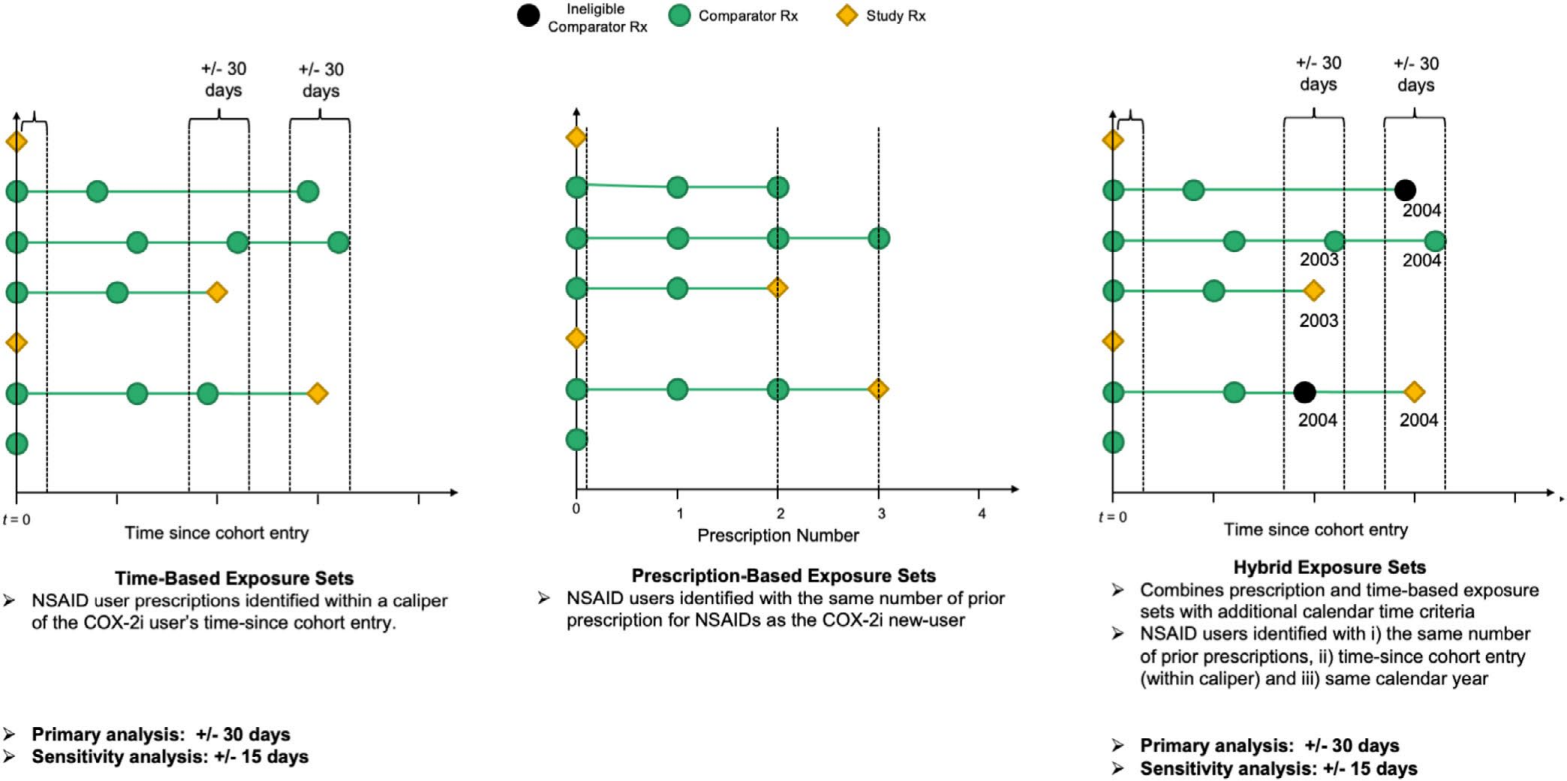
Graphical depiction of the prescription‐based (Rx‐based), time‐based and hybrid exposure sets used in this study.

The generation of a PNU cohort, as proposed by Suissa et al. [3], requires four steps (Figure 1). First, a base cohort is formed containing all new‐users of the novel study treatment, new‐users of the comparator and switchers from the comparator treatment to the study treatment. Second, for each new‐user of the study treatment, an exposure set is formed containing a pool of potential comparators similar to the study treatment user with respect to prior use of the comparator treatment. Prior use may be defined in a number of ways and consider prior number of prescriptions, time since cohort entry and calendar time (or a combination of these) (Figure 2). Third, time‐conditional propensity scores (TCPS) are estimated using a logistic regression model conditional on exposure set and adjusting for covariates. Fourth, study treatment users are matched chronologically within exposure set to the comparator user with the closest TCPS to the study treatment user, without replacement. In the resulting matched cohort, estimated average treatment effects in the treated (ATT) are obtained (often in a survival analysis framework). Given the potential for effect modification by prevalent/incident new‐user status, these estimates are usually presented overall and by new‐user subgroup.

Exposure set definition is a key investigator decision and there is currently no consensus on how these should be defined or the potential robustness of results to this choice [4, 5]. Furthermore, a recent review of PNU design applications found considerable variation in practice and none of the studies included varied this definition in sensitivity analyses [5].

We implemented the PNU design in UK electronic health record (EHR) data to investigate the impact of exposure set choice on estimated treatment effects for the association between non‐steroidal anti‐inflammatory drug (NSAIDs) and cyclo‐oxygenase‐2 inhibitor (COX‐2i) use on the risk of upper GI bleeding (UGIB), considering prescription‐based, time‐based, and hybrid exposure set definitions [3, 5, 6].

## 2 Methods

### 2.1 Data Sources

We used the UK Clinical Practice Research Datalink GOLD database (July 2020 build) which consists of primary care records for approximately 9% of the UK population, including information on prescribing, signs and symptoms, diagnoses, referrals and some lifestyle factors (e.g., on smoking and alcohol consumption) [7]. For this study, we additionally linked the data with Hospital Episode Statistics (available in England only) and Office for National Statistics (ONS) mortality data. We also linked to ONS death registration data, individual‐level index of multiple deprivation (IMD) and local‐region level rural–urban classification. Scientific approval was obtained by the Independent Scientific Advisory Committee (ISAC) (Protocol 19_273).

### 2.2 Study Population and Study Design

We used a PNU design (summarised in Figure 1) and defined a study population consisting of individuals with osteoarthritis, aged 18 years or older, initiating NSAIDs or COX‐2is between 1st January 2000 and 31st December 2004. This time window was chosen to represent a period where COX‐2is were newly introduced and therefore represents an ideal application for the PNU (codelists for the NSAID and COX‐2i prescriptions considered and available during this period are included in Tables S1 and S2). Individuals were required to have at least 12 months of up‐to‐standard data available prior to base cohort entry to allow for confounder assessment and distinguishing new‐users of treatments from historical prevalent users or switchers.

This base cohort included all new‐users of NSAIDs, new‐users of COX‐2is, and prevalent new‐users of COX‐2is.

### 2.3 Exposure Sets

The index date for COX‐2i users was the prescription date denoting new‐use (incident/prevalent). For each COX‐2i user, an exposure set was formed containing a pool of potential NSAID comparator users. These considered prior use of NSAIDs in several ways, captured by the following exposure set definitions (summarised in Figure 2):

- *Prescription‐based:* exposure sets contained all NSAID comparators with the same number of prior prescriptions.
- *Time‐based:* exposure sets contained all NSAID comparators within a caliper of (±) 30 days of the corresponding COX‐2i user on the time since base cohort entry scale.
- *Hybrid:* exposure sets contained all NSAID comparators with the same number of prior prescriptions, within a caliper of ± 30 days on the time‐since base cohort entry scale and in the same calendar year as the COX‐2i user.

In each case, the index date for NSAID users refers to the date of the relevant prescription being considered within a particular exposure set.

### 2.4 Time‐Conditional Propensity Score Estimation and Matching

We estimated TCPSs using conditional logistic regression, conditional on exposure set, across the stacked set of exposure sets. The following covariates, defined where available in primary and secondary care, were included in this model:

- *Demographics:* Age, sex, IMD score rank decile, body mass index, smoking status, alcohol consumption.
- *Comorbidities/behaviours (any recording on or prior to index date):* Hypertension, chronic renal failure, inflammatory bowel disease, gastrointestinal tract tumours, coagulopathies, gastro‐oesophageal reflux disease, diabetes, heart failure, previous upper GI bleed, number of admissions to A&E in previous 6 months.
- *Medications/therapies (any recording in the 3 months prior to index date):* anticoagulants, systemic corticosteroids, proton pump inhibitors, H2 antagonists, coronary angioplasty, selective serotonin reuptake inhibitors, statins, and clopidogrel.
- *Other:* Calendar year.

Given the base cohort was anticipated to be large, we selected random samples of comparators from each exposure set to reduce the computational burden of fitting the TCPS model [3]. Deviating from Suissa et al. [3], who recommend selecting random samples of fixed numbers (e.g., 10, 20 or 100), we selected these proportional to the size of exposure set and applied weights (equal to the inverse of the sampling fraction) to recognise differences in exposure set size.

The resulting TCPS model was used to estimate TCPSs for all members of the exposure sets, not only the sampled ones.

COX‐2i users were matched in chronological order 1:1 to the NSAID user, within exposure set, with the closest TCPS [3]. Treatment effects were estimated in the resulting matched cohort (analogous to other propensity score matching methods) and, following the original proposal, we did not consider a matched analysis for example, via a stratified Cox model [3, 8].

The positivity assumption was assessed within each exposure set (containing all members not only the sampled ones) to ensure the TCPS of the COX‐2i user was within the range of TCPSs of potential NSAID comparators in the same exposure set. If this assumption was violated, the exposure set was dropped. For example, if the TCPS for the COX‐2i user was 0.75 but the range of TCPS of potential NSAID comparators ranged from 0.24 to 0.65, this exposure set was dropped.

### 2.5 Outcome and Follow‐Up

The study outcome was the first occurrence of UGIB leading to hospitalisation (any position) or death and ascertained using HES and ONS mortality data via ICD‐10 codes (available in Table S3).

In the matched cohort, index date was the date of initiation for COX‐2is and the corresponding prescription date for the matched NSAID user. Individuals were censored at the earliest of outcome occurrence, study end date, death, incident cirrhotic liver disease (given this has a strong association with UGIB), transfer out of practice, or treatment discontinuation.

Continuous treatment periods were defined as follows:

$$\begin{array}{c} 1^{\mathrm{st}}\;\text{prescription date}+\text{duration of prescription}\\ +\text{duration of}\;\mathrm{any}\;\text{successive overlapping prescriptions}\;\left(\text{of same treatment}\right)\\ +30\;\text{days}. \end{array}$$

### 2.6 Estimands

The PNU matching process aligns with estimation of ATT effects. Considering an as‐treated follow‐up approach, this relates to the following estimand: ‘among the subgroup of the study population initiating COX‐2is, what effect does that initiation and sustained use have on their UGIB risk relative to initiation/continued and sustained use of NSAIDs?’.

We conducted three analyses in three distinct study populations, each comparing COX‐2i users and NSAID comparators, considering the following estimands:

- *Overall effect of incident new‐use and switching:* What if initiators of COX‐2is had initiated or continued using NSAIDs at the time they initiated COXI‐2is?
- *Effect of switching (PNU):* What if initiators of COX‐2is who switched from NSAIDs had continued NSAIDs at the time of switching?
- *Effect of incident new‐use:* What if initiators of COX‐2is had initiated NSAIDs at the time they initiated COX‐2is?

Under an as‐treated follow‐up approach, we are interested in sustained adherence to these treatment strategies, with individuals censored at treatment discontinuation.

### 2.7 Statistical Analysis

We described the characteristics of the resulting matched PNU cohorts for each of the before and after TCPS matching for each of the exposure set definitions considered, assessing balance using standardised mean differences [8]. To generate the unmatched NSAID user group, one NSAID comparator prescription was randomly sampled from each exposure set before matching [6, 9].

Estimated hazard ratios (HR) comparing the hazard of UGIB between COX‐2i users and NSAID comparators were obtained using Cox models, applying robust standard errors. These were estimated overall and separately in the incident and PNU subgroups [3, 10].

In sensitivity analyses, we investigated the impact of a narrower 15‐day caliper for the time‐based and hybrid exposure set approaches.

All analyses were conducted using R Statistical Software version 4.0.3. Analytical code for implementing the PNU is available at https://github.com/johntaz/PNU‐designs.

## 3 Results

### 3.1 Cohort Characteristics

The base cohort identified 100 185 individuals who received a prescription for either COX2‐is or NSAIDs. Of these, 25 742 individuals were incident new‐users of COX2‐is and 17 952 were prevalent new‐users of COX2‐is (i.e., switched from NSAID to COX2is).

Exposure sets were constructed for each COX‐2i user under the three definitions, TCPS estimated, and matching performed. We applied sampling fractions when estimating TCPSs to reduce the computational burden. We derived these to ensure that, where possible, approximately 50 NSAID prescriptions were sampled (e.g., sampling fraction for the hybrid exposure set definition was 0.00054). This reduced computational times from days to hours on a high‐performance computing cluster.

Using the hybrid exposure set definition resulted in 76% of COX‐2i users finding a match, compared to 86% under the prescription‐based and time‐based exposure set definitions. For the hybrid exposure set definition, this reflected 85% of incident new‐users and 62% of PNUs being matched; a similar pattern was seen across other exposure set definitions. The primary reason for excluding COX‐2i users was the lack of a suitably comparable NSAID user (the positivity assumption is assessed within the exposure set) as opposed to ineligibility at the time of matching (e.g., if they had a history of exclusion criteria, they are excluded outright from any further selection [3]).

Table 1 shows the characteristics of the cohort obtained under the hybrid exposure set definition. The unmatched NSAID users are slightly younger, have a lower prevalence of comorbidities, and lower recent burden of medication use compared to the COX‐2i users. After TCPS matching, good balance is achieved between the treatment groups with absolute standardised mean differences < 0.1.

**TABLE 1:** Characteristics of NSAID and COX‐2 inhibitor users in unmatched, matched samples using Hybrid exposure‐set definition with a 30‐day caliper. The unmatched NSAID user group was generated by sampling one NSAID comparator prescription from each exposure set before matching.

	Unmatched	Matched		
	NSAID	NSAID	COX‐2i	Absolute standardised differences
N	33 065	33 065	33 065	
Age (Mean (SD))	64.95 (12.83)	69.64 (11.85)	69.58 (11.91)	0.005
Male	13 512 (40.9)	10 543 (31.9)	10 625 (32.1)	0.005
Urban	5144 (15.6)	5643 (17.1)	5794 (17.5)	0.012
IMD				0.023
1	3806 (11.5)	3713 (11.2)	3786 (11.5)	
2	3790 (11.5)	3605 (10.9)	3620 (10.9)	
3	3791 (11.5)	3830 (11.6)	3689 (11.2)	
4	3818 (11.5)	3745 (11.3)	3723 (11.3)	
5	3627 (11.0)	3672 (11.1)	3805 (11.5)	
6	3306 (10.0)	3449 (10.4)	3372 (10.2)	
7	3065 (9.3)	3101 (9.4)	3184 (9.6)	
8	3060 (9.3)	3095 (9.4)	3110 (9.4)	
9	2346 (7.1)	2411 (7.3)	2337 (7.1)	
10	2456 (7.4)	2444 (7.4)	2439 (7.4)	
Hospital admissions				0.023
0	28 830 (87.2)	28 337 (85.7)	28 103 (85.0)	
1	3200 (9.7)	3560 (10.8)	3794 (11.5)	
2	698 (2.1)	768 (2.3)	783 (2.4)	
> 2	337 (1.0)	400 (1.2)	385 (1.2)	
Alcohol consumption				0.007
High	533 (1.6)	431 (1.3)	457 (1.4)	
Low	16 260 (49.2)	17 462 (52.8)	17 424 (52.7)	
Missing	16 272 (49.2)	15 172 (45.9)	15 184 (45.9)	
Smoking status				0.01
Current	5109 (15.5)	4512 (13.6)	4513 (13.6)	
Ex	6460 (19.5)	6586 (19.9)	6518 (19.7)	
Non‐smoker	15 888 (48.1)	16 777 (50.7)	16 730 (50.6)	
Missing	5608 (17.0)	5190 (15.7)	5304 (16.0)	
Body mass index				0.015
< 18.5	223 (0.7)	266 (0.8)	261 (0.8)	
18.5–25	9117 (27.6)	8736 (26.4)	8924 (27.0)	
25–30	11 030 (33.4)	11 076 (33.5)	10 957 (33.1)	
30 +	5900 (17.8)	6335 (19.2)	6233 (18.9)	
Missing	6795 (20.6)	6652 (20.1)	6690 (20.2)	
Comorbidities				
IBD	256 (0.8)	324 (1.0)	330 (1.0)	0.002
Heart failure	1049 (3.2)	1611 (4.9)	1683 (5.1)	0.01
Hypertension	10 823 (32.7)	13 184 (39.9)	13 119 (39.7)	0.004
GI cancer	328 (1.0)	385 (1.2)	387 (1.2)	0.001
CKD	136 (0.4)	155 (0.5)	165 (0.5)	0.004
Diabetes	2505 (7.6)	2784 (8.4)	2888 (8.7)	0.011
Coronary angioplasty	198 (0.6)	189 (0.6)	217 (0.7)	0.011
Coagulopathy	162 (0.5)	196 (0.6)	204 (0.6)	0.003
Previous UGIB	710 (2.1)	1025 (3.1)	1105 (3.3)	0.014
GERD	1196 (3.6)	1760 (5.3)	1834 (5.5)	0.01
Medications/therapies				
Statin	3868 (11.7)	4331 (13.1)	4434 (13.4)	0.009
PPI/H2RA	3175 (9.6)	5972 (18.1)	6032 (18.2)	0.005
SSRI	1477 (4.5)	1830 (5.5)	1877 (5.7)	0.006
Anticoagulant	310 (0.9)	471 (1.4)	515 (1.6)	0.011
Antiplatelets	5307 (16.1)	6621 (20.0)	6770 (20.5)	0.011
OCS	813 (2.5)	1331 (4.0)	1351 (4.1)	0.003
Other respiratory	2923 (8.8)	3695 (11.2)	3740 (11.3)	0.004
Calendar Year				< 0.001
2000	2749 (8.3)	2989 (9.0)	2989 (9.0)	
2001	5464 (16.5)	6147 (18.6)	6147 (18.6)	
2002	7776 (23.5)	8817 (26.7)	8817 (26.7)	
2003	8775 (26.5)	8684 (26.3)	8684 (26.3)	
2004	8301 (25.1)	6428 (19.4)	6428 (19.4)	

Abbreviations: ASD, absolute standardised differences; BMI, body mass index; CKD, chronic kidney disease; GERD, gastroesophageal reflux disease; H2RA, h2 receptor antagonists; IBD, inflammatory bowel disease; IMD, index of multiple deprivation; OCS, oral corticosteroids; PPI, proton pump inhibitor; SD, standard deviation; SSRI, Selective serotonin reuptake inhibitors; UGIB, upper gastrointestinal bleeding.

The equivalent tables for the prescription and time‐based exposure set definitions are presented in Tables S4 and S5. These cohorts resulted in a similar distribution of characteristics between the NSAID and COX‐2i users. However, there were slight residual imbalances after matching in calendar year.

In the matched cohort under the hybrid exposure set definition, 97 (0.3%) of the COX‐2i users had an incident UGIB compared to 60 (0.2%) of the NSAID users. Similar patterns were observed in the time‐based (112 vs. 72 UGIB events) and prescription‐based (114 vs. 75 UGIB events) matched cohorts.

### 3.2 Treatment Effects

**FIGURE 3:**
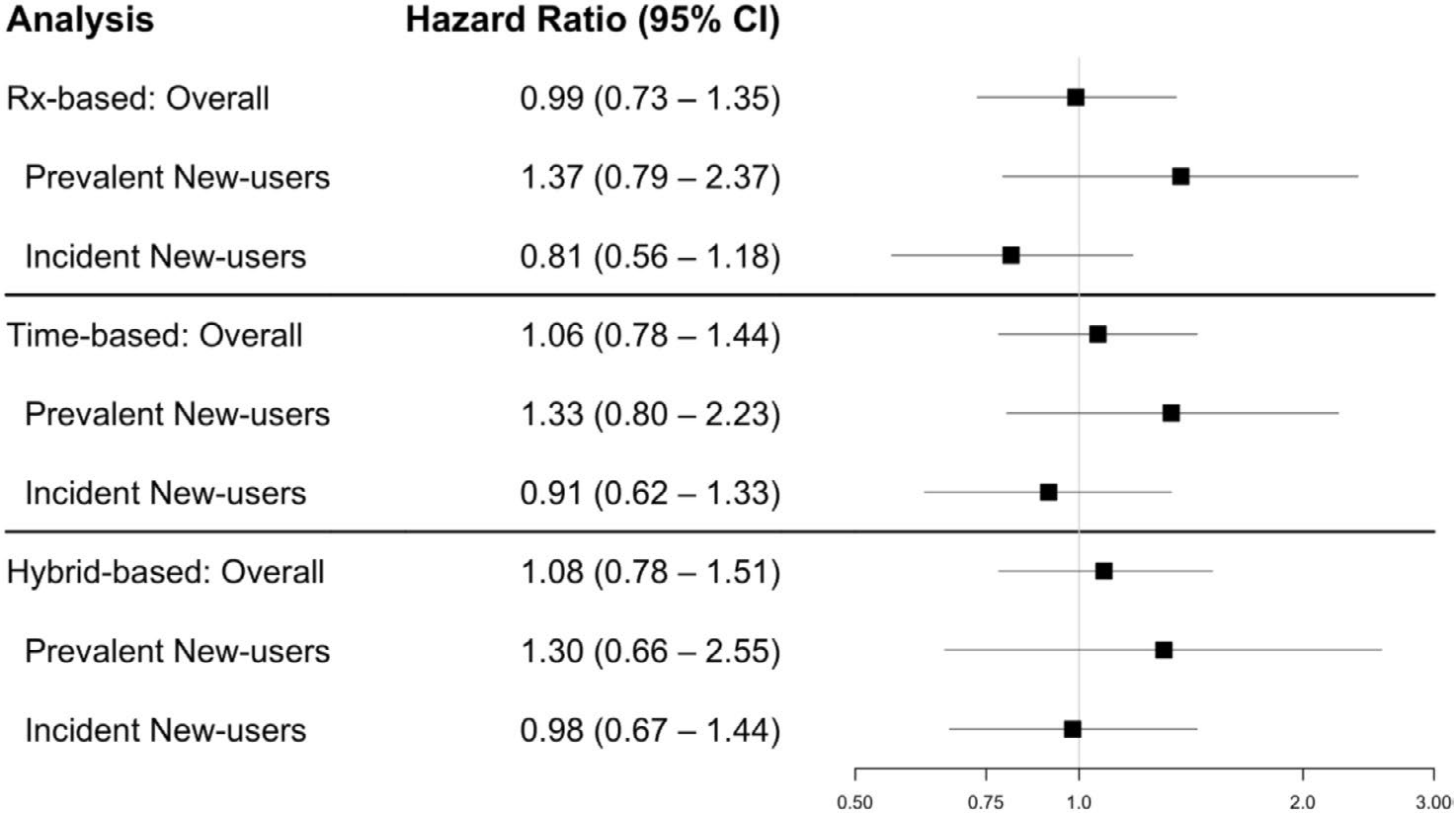
Forest plot of estimated hazard ratios for association between COX2‐i versus NSAID use and UGIB from prescription‐based (Rx‐based), time‐based and hybrid exposure set definitions. Three estimands are presented: (i) Effect of incident new‐use or switching (Overall), (ii) Effect of prevalent new‐use (i.e., switching) and (iii) Effect of incident new‐use.

In the matched cohorts, treatment effects were estimated using Cox models (summarised in Table 2 and Figure 3).

**TABLE 2:** Estimated hazard ratios comparing the hazard of UGIB in COX‐2i versus NSAID users, in matched samples using prescription (Rx), time and hybrid exposure‐set definitions.

	Overall		Subgroup: prevalent new‐users		Subgroup: incident new‐users	
	Hazard ratio (95% CI)	Confidence limit ratio	Hazard ratio (95% CI)	Confidence limit ratio	Hazard ratio (95% CI)	Confidence limit ratio
Analysis						
Rx‐based	0.99 (0.73–1.35)	1.85	1.37 (0.79–2.37)	3.00	0.81 (0.56–1.18)	2.11
Time‐based	1.06 (0.78–1.44)	1.85	1.33 (0.80–2.23)	2.79	0.91 (0.62–1.33)	2.15
Hybrid‐based	1.08 (0.78–1.51)	1.94	1.30 (0.66–2.55)	3.86	0.98 (0.67–1.44)	2.15
Sensitivity analysis						
Time‐based (15‐days)	1.07 (0.79–1.45)	1.84	1.82 (1.04–3.21)	3.09	0.81 (0.56–1.17)	2.09
Hybrid‐based (15‐days)	1.07 (0.77–1.48)	1.92	1.24 (0.64–2.38)	3.72	1.00 (0.68–1.47)	2.16

Abbreviations: CI, confidence interval; UGIB, upper gastrointestinal bleeding.

Using the hybrid exposure set definition, the overall hazard of UGIB was 8% higher in those initiating or switching to COX‐2is compared to those initiating or continuing NSAIDs (HR: 1.08, 95% CI: 0.78–1.51). The estimated HR in the subgroup of incident new‐users comparing initiators of COX‐2is to initiators of NSAIDs was 0.98 (95% CI: 0.67–1.44). In the subgroup of prevalent new‐users, prevalent new‐users of COX‐2is had a 30% increased hazard of UGIB compared to continuers of NSAIDs (HR: 1.30, 95% CI: 0.66–2.55).

Using the prescription‐based exposure set definition, the overall hazard of UGIB was 1% lower in those initiating or switching to COX‐2is compared to those initiating or continuing NSAIDs (HR: 0.99, 95% CI: 0.73–1.35). The estimated HR in the subgroup of incident new‐users comparing initiators of COX‐2is to initiators of NSAIDs was 0.81 (95% CI: 0.56–1.18). In the subgroup of prevalent new‐users, the estimated HR was 1.37 (95% CI: 0.79–2.37).

Using the time‐based exposure set definition, the overall HR of UGIB in those initiating or switching to COX‐2is compared to those initiating or continuing NSAIDs was 1.06 (95% CI: 0.78–1.44). The estimated HR in the subgroup of incident new‐users comparing initiators of COX‐2is to initiators of NSAIDs was 0.91 (95% CI: 0.62–1.33). The estimated HR in the subgroup of prevalent new‐users was 1.33 (95% CI: 0.80–2.23).

Overall, we observed some variability in the point estimates obtained under the different exposure set definitions, especially for the incident new‐user subgroup. However, the conclusions across these approaches are largely consistent. We observed similar variances for the estimated treatment effects across the approaches. Increased variance for the overall and prevalent new‐users effects was observed under the hybrid exposure definition (where a lower proportion of PNUs were matched).

### 3.3 Sensitivity Analysis

In sensitivity analyses, we investigated the impact of specifying a narrower time since cohort entry caliper for the time and hybrid exposure sets, varying this from 30 to 15 days.

These exposure definitions had a minimal impact on the number of COX‐2i users successfully matched (75% and 83% for hybrid and time exposure sets, respectively). We also observed a similar distribution of characteristics across the unmatched and matched groups compared to the 30‐day definition (Tables S6 and S7).

The pattern of estimated treatment effects was consistent with the 30‐day definition, however, there was variation in the point estimates (Figure S1). For example, using the 15‐day caliper for the time‐based exposure sets the results were, as follows. The overall HR of UGIB in those initiating or switching to COX‐2is compared to those initiating or continuing NSAIDs was 1.07 (95% CI: 0.79–1.45). The estimated HR in the subgroup of incident new‐users comparing initiators of COX‐2is to initiators of NSAIDs was 0.81 (95% CI: 0.56–1.17). The estimated HR in the subgroup of PNUs was 1.82 (95% CI: 1.04–3.21).

Finally, given the minimal differences in sample sizes, comparable variances were observed (Table 2).

## 4 Discussion

In this study, we empirically compared different exposure set definitions within the PNU design. Prescription‐based, time‐based and hybrid exposure set definitions were applied to a study investigating the association between NSAID and COX‐2i use on the risk of UGIB. Across the definitions, a similar proportion of COX‐2i users were successfully matched, with slightly fewer individuals matched by the more restrictive hybrid definition (76% vs. 86% in the time/prescription‐based). Whilst we observed consistent distributions of characteristics across the resulting matched cohorts, there were some residual imbalances in calendar year in the time‐based and prescription‐based cohorts. Overall, in this example, the pattern of results obtained was robust to the choice of exposure set definition; however, we did observe variation in the point estimates obtained under the different definitions in both primary and sensitivity analyses. Our findings highlight several considerations when deciding on exposure set definition in PNU designs.

Firstly, whilst the potential computational burden of the TCPS matching procedure can be alleviated by implementing more restrictive exposure set definitions [3, 11], this may need to be balanced with other considerations. For example, the hybrid approach was the most restrictive approach we considered and resulted in improved covariate balance compared to the prescription‐based and time‐based approaches. Although the resulting proportion of potential COX‐2i users successfully matched was 10% lower than the alternatives. In this study, this did not dramatically impact statistical power. However, since the PNU design is often applied in the setting of a novel treatment being introduced, this could importantly impact sample size and precision of estimated treatment effects in other contexts.

Another key component to consider is the role of calendar time in the exposure set definition. We found residual imbalances in calendar time of treatment initiation in the prescription and time‐based cohorts. This was also observed in a recent study by Young et al. [10], which applied prescription‐based exposure sets. In the resulting matched cohorts, this may have contributed to differences in the point estimates obtained. Importantly, given the settings PNU designs are naturally suited to, there may be important changes related to calendar time, for example, in prescribing patterns and practices, occurring during the study period. Therefore, it will often be desirable to explicitly include calendar time in the exposure set definition. This may also improve comparability between PNU designs and alternative designs, such as the sequential trial design [12], where calendar time is matched on through the spacing of successive trials, for example, in a study comparing these two designs Suissa et al. [13] generated monthly trials.

Sensitivity analyses on exposure set definition should be more routinely conducted in applications of the PNU design. For example, as highlighted in a recent review, exposure set definitions are increasingly moving away from these initial proposals to consider more complex exposure set definitions [5]. Filion and Yu [4], highlight that it may be desirable to match on other variables, for example, related to usage of related medications, to increase comparability between treatment groups. However, the potential impact of more restrictive exposure set definitions on the resulting characteristics of the matched cohort is unclear. Future work could explore how best to minimise concerns surrounding a loss of representativeness with respect to a target population of interest. This also relates to the current practice of constructing an unmatched comparison group through random sampling, which does not fully capture potential differences between the base cohort and resulting matched cohort [5]. This highlights an important gap in our understanding of how best to performance cohort diagnostics in PNU designs.

Finally, we have made the analytical code for our implementation of the PNU design in R available via GitHub. Given the absence of available software for the design currently [5], we hope this can contribute to further applications and development of the design. Computational burden can be a challenge when implementing PNU designs and will be exacerbated depending on many factors, including the number of exposure sets, potential number of comparator prescriptions, and computing resources available. In this study, with a base cohort of > 100 000 individuals, we found application of sampling fractions to be a promising solution which considerably reduced computational times.

Our study has some limitations to consider. First, we focused on comparing exposure set definitions in the context of the original TCPS matching proposal by Suissa et al. [3]. Future work could look at the impact of different TCPS matching approaches (e.g., through consideration of calipers) or using the alternative analytical strategies suggested by Webster‐Clark et al., for example using standardised morbidity ratio weighting or disease risk scores [11].

Second, the estimates obtained are not consistent with robust trial evidence suggesting a substantially lower rate of UGIB with COX‐2is compared to NSAIDs; for example, one meta‐analysis estimated a 61% relative risk reduction for clinically significant peptic ulcers [14]. In healthcare database studies, difficulties identifying subtle risk factors of UGIB, the hypothesised mechanism for residual confounding in this case study, are well documented and often require use of advanced methods for confounder adjustment, for example, high‐dimensional propensity scores [15].

Our study also consistently observed a more harmful association amongst the PNU subgroup, highlighting potential challenges around the inclusion of these patients in the analysis. These differences in the direction of effect compared with the incident new‐user subgroup are unlikely to be causal and may highlight different confounding structures between incident and prevalent new‐users [10]. The decision to switch treatment is likely multifactorial, encompassing an individual's health status, the impact of a disease and potential side‐effects of treatments on their day‐to‐day lives, the preferences of health care professional and other health‐ and non‐health‐related factors, some of which are difficult to capture in EHR databases. Another possible interpretation is that the threshold for changing an ongoing medication schedule, that is, by informing a patient that the current therapy must be switched, is likely higher than the threshold for choosing to initiate the new study drug over the older comparator for a new condition. Relatedly, the event compelling the decision to switch may also be prompted by early signs or symptoms of the outcome of interest. Finally, differences may also be partly due to the combination of different types of PNUs included in our study [16]. However, the group included are realistically reflective of the variety of patient pathways seen in clinical practice.

Consistent with many applications of the PNU design, we conducted an ‘as‐treated’ analysis [5], which has the potential to induce selection bias if censoring is informative [17]. Implementation of potential solutions, for example, via inverse probability weighting, has not been widely considered in the context of PNU designs where additional complexities, for example, relating to different mechanisms of censoring between incident and prevalent new‐users of treatment, may need to be handled.

Our study spans the introduction of the quality of outcomes framework (QOF) to UK primary care in 2004 [18]. This may lead to differential recording of certain incentivised comorbidities or other directly or indirectly QOF‐induced changes occurring in the latter part of the study period. However, choosing a period where COX‐2is were less established in the UK (they were first introduced in 1999) represented a clinical scenario where the PNU design was clearly indicated. Finally, we were unable to include dosage‐based criteria in our exposure set definitions given concerns around data quality and completeness; these may be valuable to consider in settings where suitable data are available.

## 5 Conclusion

When applying the PNU designs careful consideration should be given to exposure set definition, particularly in relation to the balance between complexity and computational efficiency and handling of calendar time. Where uncertainties in the optimal exposure set definition exist, this should be transparently explored in sensitivity analyses assessing the impact of these choices on the resulting characteristics of the matched cohorts and pattern of treatment effects.

## Funding

JT was supported by a Medical Research Council PhD Studentship (MR/N013638/1) and the Wellcome Trust (grant 2244485/Z/21/Z). EJW was funded by the Wellcome Trust (grant 224485/Z/21/Z). IJD was funded by an unrestricted grant from GSK.

## Ethics Statement

Scientific approval was obtained to use CPRD data by the Independent Scientific Advisory Committee (ISAC) (Protocol 19273) and ethical approval from the London School of Hygiene & Tropical Medicine ethics committee.

## Conflicts of Interest

DCG and IAG are employees of, and own shares in, GSK. JL was an employee of GSK at time of study implementation and owns shares in, GSK. MC was an employee of GSK at time of study implementation. IJD is funded by an unrestricted grant from, and holds stock in, GSK.

## Supporting information


**Data S1:** pds70339‐sup‐0001‐Supinfo.pdf.

## Data Availability

The analytical code for this study is provided at the following GitHub repository: https://github.com/johntaz/PNU‐designs.
