# Supplementary material for: Empirical Comparison of Exposure Set Definitions in the Prevalent New‐User Design
Source: Pharmacoepidemiol Drug Saf. 2026 Feb 27;35(3):e70339. doi: 10.1002/pds.70339 (PMC12948652; doi:10.1002/pds.70339)

Supporting Information

Tables

S1 - Code list for COX-2 inhibitors

Prodcode	Gemscript code	Product name
474	79023020	Celecoxib 100mg capsules
5080	81598020	Celebrex 200mg capsules (Pfizer Ltd)
5175	81597020	Celebrex 100mg capsules (Pfizer Ltd)
5254	79024020	Celecoxib 200mg capsules
43616	98663020	Celecoxib 400mg capsules
50059	8520020	Celebrex 100mg capsules (Necessity Supplies Ltd)
55582	8512020	Celebrex 200mg capsules (Lexon (UK) Ltd)
66757	8508020	Celebrex 200mg capsules (Waymade Healthcare Plc)
3311	60932020	Etodolac 200mg capsules
4368	58036020	Lodine 200mg Capsule (Shire Pharmaceuticals Ltd)
8969	58038020	Lodine 300mg Capsule (Shire Pharmaceuticals Ltd)
10033	60934020	Etodolac 300mg capsules
24356	83390020	Eccoxolac 300mg capsules (Meda Pharmaceuticals Ltd)
66323	86577020	Ebretin 300mg capsules (Ranbaxy (UK) Ltd)
5266	75111020	Lodine sr 600mg Modified-release tablet (Shire Pharmaceuticals Ltd)
5455	75114020	Etodolac 600mg modified-release tablets
35653	91953020	Etopan XL 600mg tablets (Sun Pharmaceuticals UK Ltd)
38770	95557020	Lodine SR 600mg tablets (Almirall Ltd)
52714	8117020	Etodolac 600mg modified-release tablets (Alliance Healthcare (Distribution) Ltd)
71908	70658021	Etolyn 600mg modified-release tablets (Mylan)
76419	75667020	Etodolac sr 600mg Tablet (Winthrop Pharmaceuticals Ltd)
8451	60933020	Etodolac 200mg Tablet
16194	58037020	Lodine 200mg Tablet (Shire Pharmaceuticals Ltd)
20386	60937020	Ramodar 200mg Tablet (Wyeth Pharmaceuticals)
650	77851020	Etoricoxib 60mg tablets
5812	77854020	Etoricoxib 90mg tablets
5938	84667020	Etoricoxib 120mg tablets
6464	84678020	Arcoxia 60mg tablets (Grunenthal Ltd)
6498	77845020	Arcoxia 90mg tablets (Grunenthal Ltd)
9822	77848020	Arcoxia 120mg tablets (Grunenthal Ltd)
37562	94524020	Arcoxia 30mg tablets (Grunenthal Ltd)
37587	94522020	Etoricoxib 30mg tablets
51284	10595020	Arcoxia 60mg tablets (Sigma Pharmaceuticals Plc)
51874	39400020	Arcoxia 30mg tablets (Lexon (UK) Ltd)
53576	10607020	Arcoxia 120mg tablets (DE Pharmaceuticals)
62658	10606020	Arcoxia 120mg tablets (Waymade Healthcare Plc)

62843	10603020	Arcoxia 90mg tablets (Lexon (UK) Ltd)
74952	10592020	Arcoxia 60mg tablets (Waymade Healthcare Plc)
75549	77465021	Etoricoxib 30mg tablets (Accord Healthcare Ltd)
7118	88358020	Prexige 100mg tablets (Novartis Pharmaceuticals UK Ltd)
10212	88352020	Lumiracoxib 100mg tablets
28171	88356020	Lumiracoxib 400mg tablets
28383	88362020	Prexige 400mg tablets (Novartis Pharmaceuticals UK Ltd)
76595	36645020	Meloxicam 7.5mg/5ml oral suspension
57370	17125021	Meloxicam 15mg orodispersible tablets sugar free
57475	17127021	Meloxicam 7.5mg orodispersible tablets sugar free
850	81638020	Mobic 7.5mg tablets (Boehringer Ingelheim Ltd)
1469	81615020	Meloxicam 15mg tablets
1470	81639020	Mobic 15mg tablets (Boehringer Ingelheim Ltd)
2243	81614020	Meloxicam 7.5mg tablets
35935	72118020	Meloxicam 7.5mg tablets (Somex Pharma)
56275	72217020	Meloxicam 7.5mg tablets (Teva UK Ltd)
66364	72791020	Meloxicam 15mg tablets (Actavis UK Ltd)
76191	72175020	Meloxicam 7.5mg tablets (A A H Pharmaceuticals Ltd)
77260	72221020	Meloxicam 15mg tablets (Teva UK Ltd)
28190	!8505504	VIOXX
28193	!8505505	VIOXX
32362	!8505522	ROFECOXIB
36669	!8505521	ROFECOXIB
613	76748020	Vioxx 12.5mg/5ml oral suspension (Merck Sharp & Dohme Ltd)
637	83963020	Rofecoxib 25mg/5ml oral suspension sugar free
640	79862020	Rofecoxib 12.5mg/5ml oral suspension sugar free
5739	59878020	Vioxx 25mg/5ml oral suspension (Merck Sharp & Dohme Ltd)
518	79860020	Rofecoxib 12.5mg tablets
538	76746020	Vioxx 12.5mg tablets (Merck Sharp & Dohme Ltd)
666	76747020	Vioxx 25mg tablets (Merck Sharp & Dohme Ltd)
706	79861020	Rofecoxib 25mg tablets
5695	80118020	VioxxAcute 50mg tablets (Merck Sharp & Dohme Ltd)
5841	81743020	Rofecoxib 50mg tablets
6460	78899020	VioxxAcute 25mg tablets (Merck Sharp & Dohme Ltd)
723	77194020	Valdecoxib 10mg tablets
6663	79996020	Valdecoxib 20mg tablets
9899	85809020	Bextra 10mg tablets (Pfizer Ltd)
9912	85182020	Bextra 20mg tablets (Pfizer Ltd)
9978	49090020	Bextra 40mg tablets (Pfizer Ltd)
18066	83422020	Valdecoxib 40mg tablets

S2 - Code list for NSAIDs

Prodcode	Gemscript code	Product name
526	81068020	Aceclofenac 100mg tablets
344	73771020	Acemetacin 60mg capsules
55099	86649020	Acoflam 100mg Retard tablets (Mercury Pharma Group Ltd)
40086	86647020	Acoflam 50mg gastro-resistant tablets (Mercury Pharma Group Ltd)
75442	86651020	Acoflam 75mg SR tablets (Mercury Pharma Group Ltd)
25257	83095020	Advil 200mg tablets (Wyeth Consumer Healthcare)
40394	83096020	Advil 400mg Tablet (Wyeth Consumer Healthcare)
32704	86005020	Advil cold and sinus 200mg+30mg Tablet (Wyeth Consumer Healthcare)
13347	48091020	Alrheumat 50mg Capsule (Bayer Plc)
32509	73886020	Anadin Ibuprofen 200mg tablets (Pfizer Consumer Healthcare Ltd)
38493	94461020	Anadin Joint Pain 200mg tablets (Pfizer Consumer Healthcare Ltd)
46860	99382020	Anadin LiquiFast 200mg effervescent tablets (Pfizer Consumer Healthcare Ltd)
43456	96457020	Anadin LiquiFast 400mg capsules (Pfizer Consumer Healthcare Ltd)
40516	96455020	Anadin Ultra 200mg capsules (Pfizer Consumer Healthcare Ltd)
37253	91330020	Anadin ultra double strength 400mg Capsule (Wyeth Consumer Healthcare)
20978	77321020	Anadin Ultra liquid capsules (Wyeth Consumer Healthcare)
31482	57638020	Apsifen 200mg Tablet (Approved Prescription Services Ltd)
27968	57639020	Apsifen 400mg Tablet (Approved Prescription Services Ltd)
31469	57645020	Apsifen -f 600mg Tablet (Approved Prescription Services Ltd)
19036	62435020	Arthrofen 200 tablets (Ashbourne Pharmaceuticals Ltd)
15068	62436020	Arthrofen 400 tablets (Ashbourne Pharmaceuticals Ltd)
21815	62437020	Arthrofen 600 tablets (Ashbourne Pharmaceuticals Ltd)
21840	62238020	Arthrosin 250 tablets (Ashbourne Pharmaceuticals Ltd)
20385	62239020	Arthrosin 500 tablets (Ashbourne Pharmaceuticals Ltd)
25341	86551020	Arthrosin EC 250 tablets (Ashbourne Pharmaceuticals Ltd)
25342	86553020	Arthrosin EC 500 tablets (Ashbourne Pharmaceuticals Ltd)
162	74346020	Arthrotec 50 gastro-resistant tablets (Pfizer Ltd)
50269	8107020	Arthrotec 75 gastro-resistant tablets (Mawdsley-Brooks & Company Ltd)
2387	81620020	Arthrotec 75 gastro-resistant tablets (Pfizer Ltd)
30168	62234020	Arthroxen 250mg Tablet (C P Pharmaceuticals Ltd)
23121	62235020	Arthroxen 500mg Tablet (C P Pharmaceuticals Ltd)

41366	97646020	Axorid 100mg/20mg modified-release capsules (Meda Pharmaceuticals Ltd)
41365	97648020	Axorid 200mg/20mg modified-release capsules (Meda Pharmaceuticals Ltd)
4049	60168020	Azapropazone 300mg capsules
3262	60169020	Azapropazone 600mg tablets
71584	60687021	Boots Ibuprofen 3 Months Plus 100mg/5ml oral suspension strawberry (The Boots Company Plc)
76093	8186020	Boots Ibuprofen 6 Months Plus 100mg/5ml oral suspension strawberry (The Boots Company Plc)
69285	60686021	Boots Ibuprofen and Codeine 200mg/12.8mg tablets (The Boots Company Plc)
71779	60688021	Boots Ibuprofen Long Lasting 200mg capsules (The Boots Company Plc)
48568	14108020	Boots Rapid Ibuprofen lysine 342mg tablets (The Boots Company Plc)
10169	77261020	Brexidol 20mg tablets (Chiesi Ltd)
19537	!0838201	BRUFEN
19538	!0838101	BRUFEN
50117	39329020	Brufen 100mg/5ml syrup (Lexon (UK) Ltd)
53397	38583020	Brufen 100mg/5ml syrup (Mawdsley-Brooks & Company Ltd)
360	48494020	Brufen 100mg/5ml syrup (Mylan)
1621	48493020	Brufen 200mg tablets (Abbott Laboratories Ltd)
1739	53998020	Brufen 400mg tablets (Mylan)
50314	8169020	Brufen 600mg effervescent granules sachets (DE Pharmaceuticals)
407	68366020	Brufen 600mg effervescent granules sachets (Mylan)
4216	54001020	Brufen 600mg tablets (Mylan)
74806	8166020	Brufen Retard 800mg tablets (Dowelhurst Ltd)
39019	95831020	Brufen Retard 800mg tablets (Mylan)
2129	68365020	Brufen retard tabs 800mg Modified-release tablet (Abbott Laboratories Ltd)
167	65704020	Butacote 100mg gastro-resistant tablets (Novartis Pharmaceuticals UK Ltd)
7483	65708020	Butazolidin 100mg Tablet (Novartis Pharmaceuticals UK Ltd)
29674	65709020	Butazolidin 200mg Tablet (Novartis Pharmaceuticals UK Ltd)
7058	83707020	Calprofen 100mg/5ml Oral suspension (McNeil Products Ltd)
49432	8177020	Calprofen 100mg/5ml oral suspension (McNeil Products Ltd)
56441	14184020	Calprofen 100mg/5ml oral suspension 5ml sachets (McNeil Products Ltd)
29316	86537020	Care ibuprofen 400mg Tablet (Thornton & Ross Ltd)
66194	49091021	Care Ibuprofen for Children 100mg/5ml oral suspension (Thornton & Ross Ltd)

7434	48703020	Clinoril 100mg tablets (Merck Sharp & Dohme Ltd)
13380	48704020	Clinoril 200mg tablets (Merck Sharp & Dohme Ltd)
28764	84951020	Closteril 100mg Modified-release tablet (Pharmalife Healthcare Services Ltd)
20036	83580020	Clotam 200mg Capsule (Thames Laboratories Ltd)
14994	85000020	Clotam Rapid 200mg tablets (Galen Ltd)
1708	54315020	Codafen Continus tablets (Napp Pharmaceuticals Ltd)
10519	4147007	CODEINE PHOS/IBUPROFEN SR (20MG/300MG) TAB
17733	86585020	Condrotec 500mg+200microgram Tablet (Pharmacia Ltd)
30389	80085020	Contraflam 250mg Capsule (Berk Pharmaceuticals Ltd)
30391	80086020	Contraflam 500mg Tablet (Berk Pharmaceuticals Ltd)
14385	50220020	Cuprofen 200mg Tablet (SSL International Plc)
37094	93812020	Cuprofen 200mg tablets (SSL International Plc)
11980	50221020	Cuprofen 400mg Tablet (SSL International Plc)
24469	73716020	Cuprofen for Children 100mg/5ml oral suspension (SSL International Plc)
39873	95907020	Cuprofen Maximum Strength 400mg tablets (SSL International Plc)
37816	87860020	Cuprofen PLUS tablets (SSL International Plc)
25362	57275020	Defanac 25mg gastro-resistant tablets (Ranbaxy (UK) Ltd)
25358	78548020	Defanac 50mg gastro-resistant tablets (Ranbaxy (UK) Ltd)
14672	75883020	Defanac 75mg SR tablets (Ranbaxy (UK) Ltd)
14707	79162020	Defanac Retard 100mg tablets (Ranbaxy (UK) Ltd)
14678	75884020	Defanac sr 100mg Modified-release tablet (Ranbaxy (UK) Ltd)
10325	89560020	Dexibuprofen 300mg tablets
11907	89572020	Dexibuprofen 400mg tablets
5173	67445020	Dexketoprofen 25mg tablets
31383	86166020	Dexomon 75mg SR tablets (Hillcross Pharmaceuticals Ltd)
16225	84262020	Dexomon retard 100mg Modified-release tablet (Hillcross Pharmaceuticals Ltd)
34744	68904020	Diclofenac 100mg Modified-release capsule (Sandoz Ltd)
27362	56927020	Diclofenac 100mg Modified-release tablet (Actavis UK Ltd)
42793	61462020	Diclofenac 100mg Modified-release tablet (IVAX Pharmaceuticals UK Ltd)
45213	92541020	Diclofenac 10mg dispersible tablets
60368	19739020	Diclofenac 10mg/5ml oral solution
61762	19741020	Diclofenac 10mg/5ml oral suspension
51808	29485020	Diclofenac 12.5mg/5ml oral solution
68849	29487020	Diclofenac 12.5mg/5ml oral suspension
73131	73411020	Diclofenac 25mg Gastro-resistant tablet (Almus Pharmaceuticals Ltd)
34362	61922020	Diclofenac 25mg Gastro-resistant tablet (Genus Pharmaceuticals Ltd)
34218	59232020	Diclofenac 25mg Gastro-resistant tablet (Pharmacia Ltd)
32536	49461020	Diclofenac 25mg Tablet (Berk Pharmaceuticals Ltd)

75136	51184020	Diclofenac 25mg Tablet (C P Pharmaceuticals Ltd)
417	72775020	Diclofenac 50mg dispersible tablets sugar free
59595	8087020	Diclofenac 50mg dispersible tablets sugar free (Sigma Pharmaceuticals Plc)
42406	73414020	Diclofenac 50mg Gastro-resistant tablet (Almus Pharmaceuticals Ltd)
33669	61923020	Diclofenac 50mg Gastro-resistant tablet (Genus Pharmaceuticals Ltd)
30297	59231020	Diclofenac 50mg Gastro-resistant tablet (Pharmacia Ltd)
54463	54642020	Diclofenac 50mg Tablet (Approved Prescription Services Ltd)
28256	49462020	Diclofenac 50mg Tablet (Berk Pharmaceuticals Ltd)
33559	51185020	Diclofenac 50mg Tablet (C P Pharmaceuticals Ltd)
30942	59698020	Diclofenac 50mg Tablet (Regent Laboratories Ltd)
64759	29493020	Diclofenac 50mg/5ml oral solution
54906	29442020	Diclofenac 50mg/5ml oral suspension
32916	68901020	Diclofenac 75mg Modified-release capsule (Sandoz Ltd)
42905	59298020	Diclofenac 75mg Modified-release tablet (Actavis UK Ltd)
30282	59264020	Diclofenac 75mg Modified-release tablet (Galen Ltd)
34212	61924020	Diclofenac 75mg Modified-release tablet (Genus Pharmaceuticals Ltd)
33645	53793020	Diclofenac 75mg Modified-release tablet (IVAX Pharmaceuticals UK Ltd)
38817	95565020	Diclofenac potassium 12.5mg tablets
628	79361020	Diclofenac potassium 25mg tablets
58572	8370020	Diclofenac potassium 25mg tablets (A A H Pharmaceuticals Ltd)
597	79362020	Diclofenac potassium 50mg tablets
43045	77025020	Diclofenac potassium 50mg tablets (Accord Healthcare Ltd)
52338	8378020	Diclofenac potassium 50mg tablets (Focus Pharmaceuticals Ltd)
1115	73894020	Diclofenac sodium 100mg modified-release capsules
72546	69323020	Diclofenac sodium 100mg modified-release capsules (A A H Pharmaceuticals Ltd)
1984	62457020	Diclofenac sodium 100mg modified-release tablets
3416	83823020	Diclofenac sodium 100mg modified-release tablets
34271	60405020	Diclofenac sodium 100mg modified-release tablets (A A H Pharmaceuticals Ltd)
649	83871020	Diclofenac sodium 25mg gastro-resistant tablets
1096	73892020	Diclofenac sodium 25mg gastro-resistant tablets
24128	51193020	Diclofenac sodium 25mg gastro-resistant tablets (A A H Pharmaceuticals Ltd)
24121	56925020	Diclofenac sodium 25mg gastro-resistant tablets (Actavis UK Ltd)
33994	53791020	Diclofenac sodium 25mg gastro-resistant tablets (IVAX Pharmaceuticals UK Ltd)

53164	68114020	Diclofenac sodium 25mg gastro-resistant tablets (Kent Pharmaceuticals Ltd)
31944	60370020	Diclofenac sodium 25mg gastro-resistant tablets (Mylan)
34091	51270020	Diclofenac sodium 25mg gastro-resistant tablets (Sandoz Ltd)
62636	60043020	Diclofenac sodium 25mg gastro-resistant tablets (Sterwin Medicines)
32108	59804020	Diclofenac sodium 25mg gastro-resistant tablets (Teva UK Ltd)
928	62455020	Diclofenac sodium 25mg tablets
1692	74349020	Diclofenac sodium 50mg gastro-resistant / Misoprostol 200microgram tablets
40	83872020	Diclofenac sodium 50mg gastro-resistant tablets
1075	73893020	Diclofenac sodium 50mg gastro-resistant tablets
26165	51194020	Diclofenac sodium 50mg gastro-resistant tablets (A A H Pharmaceuticals Ltd)
24122	56926020	Diclofenac sodium 50mg gastro-resistant tablets (Actavis UK Ltd)
34487	53792020	Diclofenac sodium 50mg gastro-resistant tablets (IVAX Pharmaceuticals UK Ltd)
27055	63521020	Diclofenac sodium 50mg gastro-resistant tablets (Kent Pharmaceuticals Ltd)
21387	60371020	Diclofenac sodium 50mg gastro-resistant tablets (Mylan)
29330	51271020	Diclofenac sodium 50mg gastro-resistant tablets (Sandoz Ltd)
31950	60044020	Diclofenac sodium 50mg gastro-resistant tablets (Sterwin Medicines)
28553	59805020	Diclofenac sodium 50mg gastro-resistant tablets (Teva UK Ltd)
917	62456020	Diclofenac sodium 50mg tablets
4880	74350020	Diclofenac sodium 75mg gastro-resistant / Misoprostol 200microgram tablets
2904	75877020	Diclofenac sodium 75mg gastro-resistant modified-release capsules
447	52256020	Diclofenac sodium 75mg modified-release capsules
32854	69320020	Diclofenac sodium 75mg modified-release capsules (A A H Pharmaceuticals Ltd)
580	83822020	Diclofenac sodium 75mg modified-release tablets
1233	62462020	Diclofenac sodium 75mg modified-release tablets
31589	51195020	Diclofenac sodium 75mg modified-release tablets (A A H Pharmaceuticals Ltd)
20653	!1857105	DICLOFENAC SODIUM S/R
20105	81306020	Dicloflex 25mg Gastro-resistant tablet (Ratiopharm UK Ltd)
40756	96608020	Dicloflex 25mg gastro-resistant tablets (Almus Pharmaceuticals Ltd)
612	50464020	Dicloflex 25mg gastro-resistant tablets (Dexcel-Pharma Ltd)

35711	92493020	Dicloflex 25mg gastro-resistant tablets (Teva UK Ltd)
9886	82072020	Dicloflex 50mg Gastro-resistant tablet (Ratiopharm UK Ltd)
39823	96610020	Dicloflex 50mg gastro-resistant tablets (Almus Pharmaceuticals Ltd)
4692	50465020	Dicloflex 50mg gastro-resistant tablets (Dexcel-Pharma Ltd)
46844	99460020	Dicloflex 75mg SR tablets (Actavis UK Ltd)
29181	91117020	Dicloflex 75mg SR tablets (Almus Pharmaceuticals Ltd)
9222	79017020	Dicloflex 75mg SR tablets (Dexcel-Pharma Ltd)
20621	84866020	Dicloflex 75mg SR tablets (Kent Pharmaceuticals Ltd)
20805	85934020	Dicloflex 75mg SR tablets (Teva UK Ltd)
35893	91115020	Dicloflex Retard 100mg tablets (Almus Pharmaceuticals Ltd)
39264	95833020	Dicloflex Retard 100mg tablets (Dexcel-Pharma Ltd)
17532	84868020	Dicloflex Retard 100mg tablets (Kent Pharmaceuticals Ltd)
42455	97700020	Dicloflex Retard 100mg tablets (Teva UK Ltd)
8789	50466020	Dicloflex retard tabs 100 100mg Modified-release tablet (Dexcel-Pharma Ltd)
17124	85935020	Dicloflex sr 100mg Tablet (IVAX Pharmaceuticals UK Ltd)
48218	629021	Dicloflex sr 100mg Tablet (Teva UK Ltd)
30790	84507020	Dicloflex sr 75mg Tablet (Genus Pharmaceuticals Ltd)
17491	76698020	Dicloflex sr 75mg Tablet (Ratiopharm UK Ltd)
3852	74400020	Diclomax 100mg Modified-release capsule (Provalis Healthcare Ltd)
74835	8102020	Diclomax Retard 100mg capsules (DE Pharmaceuticals)
38948	95891020	Diclomax Retard 100mg capsules (Galen Ltd)
74048	8101020	Diclomax Retard 100mg capsules (Mawdsley-Brooks & Company Ltd)
71362	8100020	Diclomax Retard 100mg capsules (Waymade Healthcare Plc)
71117	8046020	Diclomax SR 75mg capsules (DE Pharmaceuticals)
38881	95549020	Diclomax SR 75mg capsules (Galen Ltd)
74028	8047020	Diclomax SR 75mg capsules (Waymade Healthcare Plc)
3421	74401020	Diclomax sr 75mg Modified-release capsule (Provalis Healthcare Ltd)
9465	84244020	Diclotard 100 100mg Modified-release tablet (Galen Ltd)
9500	84230020	Diclotard 75mg modified-release tablets (Galen Ltd)
25361	86198020	Diclovol 25mg gastro-resistant tablets (Arun Pharmaceuticals Ltd)
15732	86199020	Diclovol 50mg gastro-resistant tablets (Arun Pharmaceuticals Ltd)
14084	86201020	Diclovol 75mg SR tablets (Arun Pharmaceuticals Ltd)
9688	86796020	Diclovol 75mg SR tablets (Mylan)
14085	86203020	Diclovol Retard 100mg tablets (Arun Pharmaceuticals Ltd)
27200	86798020	Diclovol Retard 100mg tablets (Mylan)

16221	57190020	Diclozip 25mg gastro-resistant tablets (Ashbourne Pharmaceuticals Ltd)
16222	57191020	Diclozip 50mg gastro-resistant tablets (Ashbourne Pharmaceuticals Ltd)
26888	85826020	Difenor xl 100mg Modified-release tablet (IVAX Pharmaceuticals UK Ltd)
18371	83511020	Digenac xl 100mg Modified-release tablet (Genus Pharmaceuticals Ltd)
48810	8229020	Dysman 250 capsules (Ashbourne Pharmaceuticals Ltd)
21831	56666020	Dysman 250mg Capsule (Ashbourne Pharmaceuticals Ltd)
13459	56667020	Dysman 500 tablets (Ashbourne Pharmaceuticals Ltd)
29587	59451020	Ebufac 400mg Tablet (DDSA Pharmaceuticals Ltd)
31787	87660020	Econac SR 75mg tablets (AMCo)
36486	87664020	Econac XL 100mg tablets (AMCo)
2258	53285020	Emflex 60mg capsules (Merck Serono Ltd)
23468	!2511301	FELDENE
341	49419020	Feldene 10mg capsules (Pfizer Ltd)
2827	55305020	Feldene 10mg dispersible tablets (Pfizer Ltd)
3935	49420020	Feldene 20 capsules (Pfizer Ltd)
7524	55306020	Feldene 20mg dispersible tablets (Pfizer Ltd)
3409	55307020	Feldene 20mg Orodispersible tablet (Pfizer Ltd)
19560	!2511302	FELDENE DISPERSIBLE
19788	!2511103	FELDENE DISPERSIBLE
39109	95883020	Feldene Melt 20mg tablets (Pfizer Ltd)
73981	8287020	Feldene Melt 20mg tablets (Sigma Pharmaceuticals Plc)
67815	8286020	Feldene Melt 20mg tablets (Waymade Healthcare Plc)
43904	98003020	Feminax Express 342mg tablets (Bayer Plc)
38511	94811020	Feminax Ultra 250mg gastro-resistant tablets (Bayer Plc)
18921	77276020	Fenactol 25mg gastro-resistant tablets (Discovery Pharmaceuticals)
17128	77273020	Fenactol 50mg gastro-resistant tablets (Discovery Pharmaceuticals)
17525	78919020	Fenactol Retard 100mg tablets (Discovery Pharmaceuticals)
17126	55714020	Fenactol SR 75mg tablets (Discovery Pharmaceuticals)
10785	53757020	Fenbid 300mg Spansules (Mercury Pharma Group Ltd)
24687	!2517102	FENBUFEN
7424	61799020	Fenbufen 300mg capsules
8145	61800020	Fenbufen 300mg tablets
14422	61806020	Fenbufen 450mg Effervescent tablet
8544	61801020	Fenbufen 450mg tablets
74641	53555020	Fenbufen 450mg tablets (A A H Pharmaceuticals Ltd)
26205	74759020	Fenbuzip 300mg Capsule (Ashbourne Pharmaceuticals Ltd)
26994	74757020	Fenbuzip 300mg Tablet (Ashbourne Pharmaceuticals Ltd)
26214	74758020	Fenbuzip 450mg Tablet (Ashbourne Pharmaceuticals Ltd)
18647	78164020	Fenoket 200mg modified-release capsules (Opus Pharmaceuticals Ltd)

4564	61812020	Fenoprofen 200mg Tablet
4469	61813020	Fenoprofen 300mg tablets
4565	61814020	Fenoprofen 600mg tablets
22158	4985007	FENOPROFEN disp 300 MG TAB
15477	2579007	FENOPRON 200 MG TAB
10678	49424020	Fenopron 300 tablets (Typharm Ltd)
10589	49425020	Fenopron 600 tablets (Typharm Ltd)
25760	5834007	FENOPRON D 300 MG TAB
18820	84269020	Fenpaed 100mg/5ml Oral suspension (Pinewood Healthcare)
65121	8176020	Fenpaed 100mg/5ml oral suspension (Pinewood Healthcare)
25800	55616020	Feverfen 100mg/5ml oral suspension (Wise Pharmaceuticals Ltd)
45814	99265020	First Resort Double Action Pain Relief 12.5mg tablets (Actavis UK Ltd)
20384	85672020	Flamatak MR 100mg tablets (Actavis UK Ltd)
20395	85673020	Flamatak MR 75mg tablets (Actavis UK Ltd)
26234	75358020	Flamatrol 10mg Capsule (Berk Pharmaceuticals Ltd)
21807	74801020	Flamrase 25 EC tablets (Teva UK Ltd)
21824	74802020	Flamrase 50 EC tablets (Teva UK Ltd)
38992	95555020	Flamrase 75mg SR tablets (Teva UK Ltd)
26212	!8504179	FLAMRASE SR
10917	74803020	Flamrase SR 100mg tablets (Teva UK Ltd)
11322	83762020	Flamrase sr 75mg Modified-release tablet (APS Berk)
71949	78255021	Flarin 200mg capsules (infirst Healthcare Ltd)
29455	84251020	Flexotard MR 100mg tablets (Pfizer Ltd)
20161	4975007	FLUFENAMIC ACID 100 MG CAP
6249	49560020	Froben 100mg tablets (Abbott Laboratories Ltd)
3182	49559020	Froben 50mg tablets (Abbott Laboratories Ltd)
38944	95551020	Froben SR 200mg capsules (Abbott Laboratories Ltd)
4043	68387020	Froben sr 200mg Modified-release capsule (Abbott Laboratories Ltd)
39354	90397020	Galpharm ibuprofen for children 100mg/5ml Oral suspension (Galpharm International Ltd)
71374	14183020	Galpharm Ibuprofen For Children 100mg/5ml oral suspension 5ml sachets (Galpharm International Ltd)
30724	86821020	Galprofen 100mg/5ml oral suspension (Galpharm International Ltd)
33785	83372020	Galprofen 200mg tablets (Galpharm International Ltd)
28888	84592020	Galprofen Long Lasting 200mg capsules (Galpharm International Ltd)
75305	93283020	Galprofen Long Lasting 300mg capsules (Galpharm International Ltd)
36597	82132020	Hedex Ibuprofen 200mg tablets (Omega Pharma Ltd)
38332	94471020	Ibucalm 200mg tablets (Aspar Pharmaceuticals Ltd)
37553	94475020	Ibucalm 400mg tablets (Aspar Pharmaceuticals Ltd)

24305	80560020	Ibufac 400mg Tablet (DDSA Pharmaceuticals Ltd)
10209	80061020	Ibufem 200mg tablets (Galpharm International Ltd)
32136	58455020	Ibular 200mg Tablet (Lagap)
76284	82706021	Ibular 200mg tablets (Ennogen Pharma Ltd)
18364	58456020	Ibular 400mg Tablet (Lagap)
76041	82708021	Ibular 400mg tablets (Ennogen Pharma Ltd)
849	74754020	Ibumed 400mg Tablet (Medipharma Ltd)
21045	59456020	Ibumetin 400mg Tablet (Alfred Benzon (UK) Ltd)
29524	63506020	Ibumetin 600mg Tablet (Alfred Benzon (UK) Ltd)
66247	65964021	Ibuprofen 100mg chewable capsules
37235	94243020	Ibuprofen 100mg/5ml / Pseudoephedrine 15mg/5ml oral suspension sugar free
647	63502020	Ibuprofen 100mg/5ml oral suspension
2938	63503020	Ibuprofen 100mg/5ml Oral suspension
29345	65371020	Ibuprofen 100mg/5ml Oral suspension (Hillcross Pharmaceuticals Ltd)
34663	68074020	Ibuprofen 100mg/5ml Oral suspension (Neo Laboratories Ltd)
48562	14177020	Ibuprofen 100mg/5ml oral suspension 5ml sachets sugar free
25205	89068020	Ibuprofen 100mg/5ml oral suspension 5ml sachets sugar free (Thornton & Ross Ltd)
48326	8170020	Ibuprofen 100mg/5ml oral suspension sugar free
33704	63505020	Ibuprofen 100mg/5ml oral suspension sugar free (A A H Pharmaceuticals Ltd)
53331	8171020	Ibuprofen 100mg/5ml oral suspension sugar free (Alliance Healthcare (Distribution) Ltd)
51828	8175020	Ibuprofen 100mg/5ml oral suspension sugar free (Kent Pharmaceuticals Ltd)
29332	62712020	Ibuprofen 100mg/5ml oral suspension sugar free (Sandoz Ltd)
52617	8184020	Ibuprofen 100mg/5ml oral suspension sugar free (Sigma Pharmaceuticals Plc)
26970	68245020	Ibuprofen 100mg/5ml oral suspension sugar free (Teva UK Ltd)
32862	71142020	Ibuprofen 100mg/5ml oral suspension sugar free (Thornton & Ross Ltd)
29352	68023020	Ibuprofen 100mg/5ml oral suspension sugar free (Vantage)
215	4856007	IBUPROFEN 200 MG CAP
11554	77460020	Ibuprofen 200mg / Codeine 12.8mg tablets
45988	97483020	Ibuprofen 200mg / Phenylephrine 5mg tablets
28522	75899020	Ibuprofen 200mg / Pseudoephedrine hydrochloride 30mg tablets
49277	8143020	Ibuprofen 200mg caplets (Bristol Laboratories Ltd)
40083	88937020	Ibuprofen 200mg caplets (Galpharm International Ltd)
51614	8135020	Ibuprofen 200mg caplets (Lloyds Pharmacy Ltd)
50266	8136020	Ibuprofen 200mg caplets (The Boots Company Plc)

61953	29732021	Ibuprofen 200mg caplets (Wockhardt UK Ltd)
586	80144020	Ibuprofen 200mg Capsule
10149	86472020	Ibuprofen 200mg capsules
59067	17816021	Ibuprofen 200mg capsules (AM Distributions (Yorkshire) Ltd)
30243	50930020	Ibuprofen 200mg effervescent tablets
75338	82223021	Ibuprofen 200mg medicated plasters
392	53037020	Ibuprofen 200mg modified-release capsules
5648	80145020	Ibuprofen 200mg orodispersible tablets sugar free
1468	71645020	Ibuprofen 200mg Soluble tablet
30382	53953020	Ibuprofen 200mg Tablet (C P Pharmaceuticals Ltd)
34911	49827020	Ibuprofen 200mg Tablet (Celltech Pharma Europe Ltd)
45331	62584020	Ibuprofen 200mg Tablet (Co-Pharma Ltd)
34621	66405020	Ibuprofen 200mg Tablet (Nucare Plc)
34931	62843020	Ibuprofen 200mg Tablet (Regent Laboratories Ltd)
416	59354020	Ibuprofen 200mg tablets
16001	49832020	Ibuprofen 200mg tablets (A A H Pharmaceuticals Ltd)
65471	69610020	Ibuprofen 200mg tablets (Almus Pharmaceuticals Ltd)
52154	67026020	Ibuprofen 200mg tablets (Galpharm International Ltd)
41513	57811020	Ibuprofen 200mg tablets (IVAX Pharmaceuticals UK Ltd)
42108	62385020	Ibuprofen 200mg tablets (OBG Pharmaceuticals Ltd)
29749	60159020	Ibuprofen 200mg tablets (Ranbaxy (UK) Ltd)
45320	59470020	Ibuprofen 200mg tablets (Sandoz Ltd)
28348	53366020	Ibuprofen 200mg tablets (Teva UK Ltd)
34447	57304020	Ibuprofen 200mg tablets (Thornton & Ross Ltd)
34354	56266020	Ibuprofen 200mg tablets (Vantage)
34527	59965020	Ibuprofen 200mg tablets (Zentiva)
60035	69002020	Ibuprofen 200mg tablets film coated (Actavis UK Ltd)
34980	53948020	Ibuprofen 200mg tablets sugar coated (Actavis UK Ltd)
48084	94572020	Ibuprofen 200mg/5ml oral suspension
75893	85432021	Ibuprofen 200mg/5ml oral suspension sugar free
28172	75901020	Ibuprofen 300mg / Pseudoephedrine 45mg modified-release capsules
11461	59563020	Ibuprofen 300mg modified-release / Codeine 20mg tablets
784	63501020	Ibuprofen 300mg modified-release capsules
48546	8156020	Ibuprofen 400mg caplets (Bristol Laboratories Ltd)
48644	8148020	Ibuprofen 400mg caplets (Lloyds Pharmacy Ltd)
50628	8149020	Ibuprofen 400mg caplets (The Boots Company Plc)
14333	91245020	Ibuprofen 400mg capsules
4911	80143020	Ibuprofen 400mg Granules
45216	53954020	Ibuprofen 400mg Tablet (C P Pharmaceuticals Ltd)
34889	49829020	Ibuprofen 400mg Tablet (Celltech Pharma Europe Ltd)
34425	53852020	Ibuprofen 400mg Tablet (Family Health)
34757	59877020	Ibuprofen 400mg Tablet (Unichem)
15	59355020	Ibuprofen 400mg tablets

19046	49833020	Ibuprofen 400mg tablets (A A H Pharmaceuticals Ltd)
57112	8146020	Ibuprofen 400mg tablets (Alliance Healthcare (Distribution) Ltd)
34536	57812020	Ibuprofen 400mg tablets (IVAX Pharmaceuticals UK Ltd)
34729	62386020	Ibuprofen 400mg tablets (OBG Pharmaceuticals Ltd)
75677	8155020	Ibuprofen 400mg tablets (Phoenix Healthcare Distribution Ltd)
46921	60160020	Ibuprofen 400mg tablets (Ranbaxy (UK) Ltd)
32875	59471020	Ibuprofen 400mg tablets (Sandoz Ltd)
27782	53365020	Ibuprofen 400mg tablets (Teva UK Ltd)
33589	57305020	Ibuprofen 400mg tablets (Thornton & Ross Ltd)
34359	56114020	Ibuprofen 400mg tablets (Vantage)
34550	69005020	Ibuprofen 400mg tablets film coated (Actavis UK Ltd)
27783	53949020	Ibuprofen 400mg tablets sugar coated (Actavis UK Ltd)
56213	8145020	Ibuprofen 400mg tablets sugar coated (Kent Pharmaceuticals Ltd)
3599	50929020	Ibuprofen 600mg effervescent granules sachets
43911	53955020	Ibuprofen 600mg Tablet (C P Pharmaceuticals Ltd)
45842	49828020	Ibuprofen 600mg Tablet (Celltech Pharma Europe Ltd)
40253	62183020	Ibuprofen 600mg Tablet (Sovereign Medical Ltd)
1086	59356020	Ibuprofen 600mg tablets
32100	49834020	Ibuprofen 600mg tablets (A A H Pharmaceuticals Ltd)
41701	53950020	Ibuprofen 600mg tablets (Actavis UK Ltd)
67740	73278020	Ibuprofen 600mg tablets (Fannin UK Ltd)
46942	55366020	Ibuprofen 600mg tablets (IVAX Pharmaceuticals UK Ltd)
34961	59472020	Ibuprofen 600mg tablets (Sandoz Ltd)
58652	8163020	Ibuprofen 600mg tablets (Sigma Pharmaceuticals Plc)
34850	53364020	Ibuprofen 600mg tablets (Teva UK Ltd)
1392	50928020	Ibuprofen 800mg modified-release tablets
2622	68109020	Ibuprofen 800mg tablets
12709	77459020	Ibuprofen and codeine 200mg + 12.5mg Tablet
49266	8183020	Ibuprofen for Children 100mg/5ml oral suspension (Galpharm International Ltd)
4309	86342020	Ibuprofen lysine 200mg tablets
54514	46754020	Ibuprofen lysine 400mg oral powder sachets
26095	90473020	Ibuprofen lysine 400mg tablets
345	3721007	IBUPROFEN S/R 300 MG CAP
39502	95773020	Ibuprofen sodium dihydrate 200mg tablets
66567	95775020	Ibuprofen sodium dihydrate 400mg tablets
76234	85433021	Ibuprofen Twelve Plus Pain Relief 200mg/5ml oral suspension (Aspire Pharma Ltd)
28822	75900020	Ibuprofen with pseudoephedrine hc 400mg + 60mg Liquid
30821	6285007	INDOPROFEN 200 MG TAB
43032	84356020	Inoven 200mg Tablet (Janssen-Cilag Ltd)
33457	68415020	Isclofen 50mg Gastro-resistant tablet (Isis Products Ltd)

25794	56702020	Isisfen 400mg Tablet (Isis Products Ltd)
30327	84738020	Jomethid XL 200mg capsules (Actavis UK Ltd)
1030	72737020	Junifen 100mg/5ml Oral suspension (Crookes Healthcare Ltd)
50652	8173020	Junior Ibuprofen 100mg/5ml oral suspension (Numark Ltd)
9637	79359020	Keral 25mg tablets (A. Menarini Farmaceutica Internazionale SRL)
15286	77485020	Ketocid 200 modified-release capsules (Chiesi Ltd)
21050	74798020	Ketonal 100mg Capsule (Lagap)
41364	97642020	Ketoprofen 100mg / Omeprazole 20mg modified-release capsules
1231	63884020	Ketoprofen 100mg capsules
40141	55793020	Ketoprofen 100mg capsules (A A H Pharmaceuticals Ltd)
46940	59918020	Ketoprofen 100mg capsules (Mylan)
1571	69511020	Ketoprofen 100mg modified-release capsules
75573	55791020	Ketoprofen 100mg modified-release capsules (A A H Pharmaceuticals Ltd)
8385	69513020	Ketoprofen 150mg modified-release capsules
41367	97644020	Ketoprofen 200mg / Omeprazole 20mg modified-release capsules
33568	57010020	Ketoprofen 200mg Modified-release capsule (Actavis UK Ltd)
46920	56131020	Ketoprofen 200mg Modified-release capsule (Generics (UK) Ltd)
3043	69512020	Ketoprofen 200mg modified-release capsules
77293	55792020	Ketoprofen 200mg modified-release capsules (A A H Pharmaceuticals Ltd)
389	63883020	Ketoprofen 50mg capsules
75581	53673020	Ketoprofen cr 100mg Capsule (Bristol-Myers Squibb Pharmaceuticals Ltd)
33180	53674020	Ketoprofen cr 200mg Capsule (Bristol-Myers Squibb Pharmaceuticals Ltd)
42500	53591020	Ketoprofen sr 100mg Capsule (Approved Prescription Services Ltd)
46919	53592020	Ketoprofen sr 200mg Capsule (Approved Prescription Services Ltd)
16637	69066020	Ketorolac 10mg tablets
29772	82614020	Ketotard XL 200mg capsules (Galen Ltd)
17818	74336020	Ketovail 100mg modified-release capsules (Teva UK Ltd)
25701	74337020	Ketovail 200mg modified-release capsules (Teva UK Ltd)
21955	53056020	Ketozip 200 XL capsules (Ashbourne Pharmaceuticals Ltd)
31962	84318020	Ketpron XL 200mg capsules (Mercury Pharma Group Ltd)
75771	69043020	Larafen 100mg Capsule (Sandoz Ltd)
32227	80610020	Larafen CR 200mg capsules (Ennogen Pharma Ltd)
7426	50135020	Lederfen 300mg Capsule (Wyeth Pharmaceuticals)
14380	84600020	Lederfen 300mg capsules (Mercury Pharma Group Ltd)
7522	50134020	Lederfen 300mg Tablet (Wyeth Pharmaceuticals)

17131	57844020	Lederfen 300mg tablets (Mercury Pharma Group Ltd)
7481	50136020	Lederfen 450mg Tablet (Wyeth Pharmaceuticals)
16176	77247020	Lederfen 450mg tablets (Mercury Pharma Group Ltd)
10481	54744020	Lederfen f 450mg Tablet (Wyeth Pharmaceuticals)
30164	88340020	Lemsip Cold and Flu Sinus 12 Hr Ibuprofen + Pseudoephedrine modified-release capsules (Reckitt Benckiser Healthcare (UK) Ltd)
22283	85582020	Lemsip flu 12 hr Modified-release capsule (Reckitt Benckiser Healthcare (UK) Ltd)
21811	59661020	Lidifen 200mg Tablet (Berk Pharmaceuticals Ltd)
21813	59662020	Lidifen 400mg Tablet (Berk Pharmaceuticals Ltd)
21821	59663020	Lidifen f 600mg Tablet (Berk Pharmaceuticals Ltd)
25329	79838020	Lofensaid 25mg gastro-resistant tablets (Opus Pharmaceuticals Ltd)
18798	79839020	Lofensaid 50mg gastro-resistant tablets (Opus Pharmaceuticals Ltd)
16272	82238020	Lofensaid Retard 100 tablets (Opus Pharmaceuticals Ltd)
16286	82237020	Lofensaid Retard 75 tablets (Opus Pharmaceuticals Ltd)
29110	83611020	Lornoxicam 4mg tablets
30122	83613020	Lornoxicam 8mg tablets
18527	86224020	Mandafen 400mg tablets (M & A Pharmachem Ltd)
30892	84841020	Mandafen for Children 100mg/5ml oral suspension sugar free (M & A Pharmachem Ltd)
36606	83343020	Manorfen 400mg tablets (The Manor Drug Company (Nottingham) Ltd)
46342	99120020	Medifen 3with months 100mg/5ml Oral suspension (SSL International Plc)
4710	49999020	Mefenamic acid 250mg Capsule (Actavis UK Ltd)
34898	49994020	Mefenamic acid 250mg Capsule (Berk Pharmaceuticals Ltd)
41677	54078020	Mefenamic acid 250mg Capsule (IVAX Pharmaceuticals UK Ltd)
46967	59436020	Mefenamic acid 250mg Capsule (Sandoz Ltd)
34924	55454020	Mefenamic acid 250mg Capsule (Teva UK Ltd)
259	59793020	Mefenamic acid 250mg capsules
34438	50007020	Mefenamic acid 250mg capsules (A A H Pharmaceuticals Ltd)
70221	8228020	Mefenamic acid 250mg capsules (Alliance Healthcare (Distribution) Ltd)
57007	76223020	Mefenamic acid 250mg capsules (Essential Generics Ltd)
46968	60540020	Mefenamic acid 250mg capsules (Mylan)
34793	60065020	Mefenamic acid 250mg capsules (Zentiva)
1983	64363020	Mefenamic acid 250mg Dispersible tablet
75154	20463020	Mefenamic acid 250mg/5ml oral suspension
34910	49995020	Mefenamic acid 500mg Tablet (Berk Pharmaceuticals Ltd)
1073	64364020	Mefenamic acid 500mg tablets
32105	50008020	Mefenamic acid 500mg tablets (A A H Pharmaceuticals Ltd)

32090	50000020	Mefenamic acid 500mg tablets (Actavis UK Ltd)
57297	8234020	Mefenamic acid 500mg tablets (Alliance Healthcare (Distribution) Ltd)
64103	78544020	Mefenamic acid 500mg tablets (Almus Pharmaceuticals Ltd)
61581	76226020	Mefenamic acid 500mg tablets (Essential Generics Ltd)
32234	54079020	Mefenamic acid 500mg tablets (IVAX Pharmaceuticals UK Ltd)
51827	8236020	Mefenamic acid 500mg tablets (Sigma Pharmaceuticals Plc)
41524	55455020	Mefenamic acid 500mg tablets (Teva UK Ltd)
34595	60066020	Mefenamic acid 500mg tablets (Zentiva)
76310	20465020	Mefenamic acid 500mg/5ml oral suspension
9736	64365020	Mefenamic acid 50mg/5ml oral suspension
20709	!4405104	MEFENAMIC ACID DISPERSIBLE
22230	78062020	Meflam 250mg Capsule (Trinity Pharmaceuticals Ltd)
26522	78063020	Meflam 500mg Tablet (Trinity Pharmaceuticals Ltd)
36260	85863020	Mendys 250mg Capsule (Kent Pharmaceuticals Ltd)
37053	82155020	Migrafen 200mg tablets (Chatfield Laboratories)
64595	16443021	Misofen 50mg/200microgram gastro-resistant tablets (Morningside Healthcare Ltd)
58842	16444021	Misofen 75mg/200microgram gastro-resistant tablets (Morningside Healthcare Ltd)
24531	69422020	Mobiflex 20mg Effervescent tablet (Roche Products Ltd)
31064	69421020	Mobiflex 20mg Granules (Roche Products Ltd)
12075	69420020	Mobiflex 20mg Tablet (Roche Products Ltd)
71152	8317020	Mobiflex 20mg tablets (Dowelhurst Ltd)
42604	98118020	Mobiflex 20mg tablets (Meda Pharmaceuticals Ltd)
8062	75820020	Motifene 75mg modified-release capsules (Daiichi Sankyo UK Ltd)
16192	56328020	Motrin 200mg Tablet (Pharmacia Ltd)
8401	56329020	Motrin 400mg tablets (Pfizer Ltd)
17201	56330020	Motrin 600mg tablets (Pfizer Ltd)
16193	68112020	Motrin 800mg tablets (Pfizer Ltd)
16474	73719020	Nabumetone 500mg dispersible tablets sugar free
2234	68000020	Nabumetone 500mg tablets
42821	61088020	Nabumetone 500mg tablets (A A H Pharmaceuticals Ltd)
13818	60823020	Nabumetone 500mg tablets (Actavis UK Ltd)
64297	62443020	Nabumetone 500mg tablets (Mylan)
11466	68001020	Nabumetone 500mg/5ml oral-suspension
19559	!4802101	NAPROSYN
23268	!4802102	NAPROSYN
4320	57758020	Naprosyn 125mg/5ml oral suspension (Roche Products Ltd)
2288	57757020	Naprosyn 250mg tablets (Atnahs Pharma UK Ltd)
34143	73699020	Naprosyn 375 Tablet (Roche Products Ltd)
19007	52497020	Naprosyn 500mg Granules (Roche Products Ltd)
1866	57763020	Naprosyn 500mg tablets (Atnahs Pharma UK Ltd)

3972	67330020	Naprosyn EC 250mg tablets (Atnahs Pharma UK Ltd)
4045	67331020	Naprosyn EC 375mg tablets (Atnahs Pharma UK Ltd)
3901	67332020	Naprosyn EC 500mg tablets (Atnahs Pharma UK Ltd)
8663	74549020	Naprosyn S/R 500mg tablets (Roche Products Ltd)
28313	!4804105	NAPROXEN
56762	31715020	Naproxen 100mg/5ml oral suspension
5407	64942020	Naproxen 125mg/5ml oral suspension
66993	68881021	Naproxen 125mg/5ml oral suspension sugar free
39693	96624020	Naproxen 200mg/5ml oral suspension
2391	4699007	NAPROXEN 250 MG CAP
65862	61301021	Naproxen 250mg effervescent tablets sugar free
34670	59135020	Naproxen 250mg Gastro-resistant tablet (Galen Ltd)
3431	74064020	Naproxen 250mg gastro-resistant tablets
34738	60413020	Naproxen 250mg gastro-resistant tablets (A A H Pharmaceuticals Ltd)
65348	40593020	Naproxen 250mg gastro-resistant tablets (Genesis Pharmaceuticals Ltd)
40401	57817020	Naproxen 250mg gastro-resistant tablets (IVAX Pharmaceuticals UK Ltd)
34289	59490020	Naproxen 250mg gastro-resistant tablets (Mylan)
34290	53361020	Naproxen 250mg gastro-resistant tablets (Teva UK Ltd)
34923	49074020	Naproxen 250mg Tablet (Berk Pharmaceuticals Ltd)
661	58922020	Naproxen 250mg tablets
39085	49087020	Naproxen 250mg tablets (A A H Pharmaceuticals Ltd)
51829	8242020	Naproxen 250mg tablets (Kent Pharmaceuticals Ltd)
53980	8247020	Naproxen 250mg tablets (Phoenix Healthcare Distribution Ltd)
54783	53359020	Naproxen 250mg tablets (Teva UK Ltd)
68685	47562020	Naproxen 250mg tablets (Waymade Healthcare Plc)
28255	49079020	Naproxen 250mg tablets (Wockhardt UK Ltd)
56554	20548020	Naproxen 250mg/5ml oral suspension
68470	68882021	Naproxen 25mg/ml oral suspension sugar free (Orion Pharma (UK) Ltd)
3432	74066020	Naproxen 375mg gastro-resistant tablets
15023	74183020	Naproxen 375mg Modified-release tablet
2197	58924020	Naproxen 375mg Tablet
44800	99218020	Naproxen 500mg / Esomeprazole 20mg modified-release tablets
46848	75583020	Naproxen 500mg Gastro-resistant tablet (Almus Pharmaceuticals Ltd)
34977	59136020	Naproxen 500mg Gastro-resistant tablet (Galen Ltd)
31945	59110020	Naproxen 500mg Gastro-resistant tablet (Sterwin Medicines)
3053	74065020	Naproxen 500mg gastro-resistant tablets
34743	49089020	Naproxen 500mg gastro-resistant tablets (A A H Pharmaceuticals Ltd)

30982	61530020	Naproxen 500mg gastro-resistant tablets (Actavis UK Ltd)
54476	40594020	Naproxen 500mg gastro-resistant tablets (Genesis Pharmaceuticals Ltd)
34610	59491020	Naproxen 500mg gastro-resistant tablets (Mylan)
27366	57207020	Naproxen 500mg gastro-resistant tablets (Teva UK Ltd)
15104	74182020	Naproxen 500mg Granules
5268	74184020	Naproxen 500mg modified-release tablets
48161	75930020	Naproxen 500mg Tablet (Almus Pharmaceuticals Ltd)
34922	49075020	Naproxen 500mg Tablet (Berk Pharmaceuticals Ltd)
46440	63631020	Naproxen 500mg Tablet (M & A Pharmachem Ltd)
807	58923020	Naproxen 500mg tablets
34769	49088020	Naproxen 500mg tablets (A A H Pharmaceuticals Ltd)
54304	57065020	Naproxen 500mg tablets (Actavis UK Ltd)
55486	53360020	Naproxen 500mg tablets (Teva UK Ltd)
39317	49080020	Naproxen 500mg tablets (Wockhardt UK Ltd)
4984	66530020	Naproxen 500mg tablets and Misoprostol 200microgram tablets
56106	20550020	Naproxen 500mg/5ml oral suspension
71709	75871021	Naproxen 50mg/ml oral suspension (A A H Pharmaceuticals Ltd)
69828	75196021	Naproxen 50mg/ml oral suspension (Alliance Healthcare (Distribution) Ltd)
69645	73768021	Naproxen 50mg/ml oral suspension (Thornton & Ross Ltd)
76955	31720020	Naproxen 75mg/5ml oral suspension
15180	86566020	Naproxen and misoprostol 500mgwith200microgram combined Tablet
45262	92261020	Naproxen Oral solution
20704	!4805101	NAPROXEN SODIUM
1043	64945020	Naproxen sodium 275mg tablets
35890	93688020	Nurofen 200mg caplets (Reckitt Benckiser Healthcare (UK) Ltd)
7535	85652020	Nurofen 200mg Capsule (Crookes Healthcare Ltd)
35292	93690020	Nurofen 200mg liquid capsules (Reckitt Benckiser Healthcare (UK) Ltd)
3597	73682020	Nurofen 200mg Soluble tablet (Crookes Healthcare Ltd)
402	85651020	Nurofen 200mg Tablet (Crookes Healthcare Ltd)
4298	73681020	Nurofen 200mg Tablet (Crookes Healthcare Ltd)
36650	93686020	Nurofen 200mg tablets (Reckitt Benckiser Healthcare (UK) Ltd)
25619	73683020	Nurofen 400mg Tablet (Crookes Healthcare Ltd)
24887	86353020	Nurofen Advance 200mg tablets (Crookes Healthcare Ltd)
28479	88706020	Nurofen Back Pain SR 300mg capsules (Reckitt Benckiser Healthcare (UK) Ltd)
72156	21548021	Nurofen Cold & Flu Relief 200mg/5mg tablets (Reckitt Benckiser Healthcare (UK) Ltd)
15363	75904020	Nurofen Cold and Flu tablets (Reckitt Benckiser Healthcare (UK) Ltd)

37002	93924020	Nurofen Express 200mg liquid capsules (Reckitt Benckiser Healthcare (UK) Ltd)
39758	95779020	Nurofen Express 256mg caplets (Reckitt Benckiser Healthcare (UK) Ltd)
42397	95785020	Nurofen Express 256mg tablets (Reckitt Benckiser Healthcare (UK) Ltd)
37731	93916020	Nurofen Express 342mg caplets (Reckitt Benckiser Healthcare (UK) Ltd)
37648	93920020	Nurofen Express 400mg liquid capsules (Reckitt Benckiser Healthcare (UK) Ltd)
44483	95783020	Nurofen Express 512mg tablets (Reckitt Benckiser Healthcare (UK) Ltd)
36787	93922020	Nurofen Express 684mg caplets (Reckitt Benckiser Healthcare (UK) Ltd)
61878	45498020	Nurofen Express Period Pain 200mg capsules (Reckitt Benckiser Healthcare (UK) Ltd)
55153	46755020	Nurofen Express Soluble 400mg oral powder sachets (Reckitt Benckiser Healthcare (UK) Ltd)
29068	91247020	Nurofen Extra Strength 400mg capsules (Reckitt Benckiser Healthcare (UK) Ltd)
73040	65965021	Nurofen for Children 100mg chewable capsules (Reckitt Benckiser Healthcare (UK) Ltd)
4731	86753020	Nurofen for children 100mg/5ml Oral suspension (Reckitt Benckiser Healthcare (UK) Ltd)
48738	8174020	Nurofen for Children 100mg/5ml oral suspension orange (Reckitt Benckiser Healthcare (UK) Ltd)
49133	8180020	Nurofen for Children 100mg/5ml oral suspension strawberry (Reckitt Benckiser Healthcare (UK) Ltd)
35265	93625020	Nurofen for children 3 months to 9 years 100mg/5ml Oral suspension (Reckitt Benckiser Healthcare (UK) Ltd)
44233	98805020	Nurofen for children baby 100mg/5ml Oral suspension (Reckitt Benckiser Healthcare (UK) Ltd)
60510	21431021	Nurofen for Children Cold, Pain and Fever Orange Flavour 100mg/5ml oral suspension (Reckitt Benckiser Healthcare (UK) Ltd)
59502	21430021	Nurofen for Children Cold, Pain and Fever Strawberry Flavour 100mg/5ml oral suspension (Reckitt Benckiser Healthcare (UK) Ltd)
51769	14178020	Nurofen for Children Singles 100mg/5ml oral suspension 5ml sachets orange (Reckitt Benckiser Healthcare (UK) Ltd)
50363	14181020	Nurofen for Children Singles 100mg/5ml oral suspension 5ml sachets strawberry (Reckitt Benckiser Healthcare (UK) Ltd)
70878	60950021	Nurofen Joint & Back Pain Relief 200mg capsules (Reckitt Benckiser Healthcare (UK) Ltd)
69018	61136021	Nurofen Joint & Back Pain Relief 256mg tablets (Reckitt Benckiser Healthcare (UK) Ltd)

22206	82951020	Nurofen Long Lasting 300mg capsules (Crookes Healthcare Ltd)
33935	90475020	Nurofen Maximum Strength Migraine Pain 684mg caplets (Reckitt Benckiser Healthcare (UK) Ltd)
11550	68069020	Nurofen Meltlets 200mg tablets (Reckitt Benckiser Healthcare (UK) Ltd)
18812	76985020	Nurofen meltlets lemon 200mg Orodispersible tablet (Reckitt Benckiser Healthcare (UK) Ltd)
23425	82652020	Nurofen Migraine Pain 342mg tablets (Reckitt Benckiser Healthcare (UK) Ltd)
13893	77456020	Nurofen Plus tablets (Reckitt Benckiser Healthcare (UK) Ltd)
28168	83270020	Nurofen Recovery 200mg orodispersible tablets (Reckitt Benckiser Healthcare (UK) Ltd)
46141	89638020	Nurofen Tension Headache 342mg caplets (Reckitt Benckiser Healthcare (UK) Ltd)
46904	130021	Nuromol 200mg/500mg tablets (Reckitt Benckiser Healthcare (UK) Ltd)
3496	74060020	Nycopren 250mg gastro-resistant tablets (Ardern Healthcare Ltd)
17165	74061020	Nycopren 500mg gastro-resistant tablets (Ardern Healthcare Ltd)
33801	78352020	Opustan 250mg Capsule (Opus Pharmaceuticals Ltd)
26247	78353020	Opustan 500mg Tablet (Opus Pharmaceuticals Ltd)
38182	94245020	Orbifen Cold & Flu oral suspension (Orbis Consumer Products Ltd)
18196	81617020	Orbifen for children 100mg/5ml Oral suspension (Orbis Consumer Products Ltd)
51943	8172020	Orbifen For Children 100mg/5ml oral suspension (Orbis Consumer Products Ltd)
11999	50920020	Orudis 100mg Capsule (Hawgreen Ltd)
40484	96899020	Orudis 100mg capsules (Sanofi)
12122	50919020	Orudis 50mg Capsule (Hawgreen Ltd)
40336	96897020	Orudis 50mg capsules (Sanofi)
71376	8218020	Oruvail 100 modified-release capsules (Mawdsley-Brooks & Company Ltd)
40215	96903020	Oruvail 100 modified-release capsules (Sanofi)
71127	8217020	Oruvail 100 modified-release capsules (Waymade Healthcare Plc)
3326	50923020	Oruvail 100mg Modified-release capsule (Hawgreen Ltd)
40664	96907020	Oruvail 150 modified-release capsules (Sanofi)
7840	50925020	Oruvail 150mg Modified-release capsule (Hawgreen Ltd)
74005	8222020	Oruvail 200 modified-release capsules (Dowelhurst Ltd)
67803	8225020	Oruvail 200 modified-release capsules (Lexon (UK) Ltd)
40185	96905020	Oruvail 200 modified-release capsules (Sanofi)
71104	8221020	Oruvail 200 modified-release capsules (Waymade Healthcare Plc)
838	50924020	Oruvail 200mg Modified-release capsule (Hawgreen Ltd)

21814	!5214001	ORUVAIL S/R
75720	71032021	Paracetamol 500mg / Ibuprofen 150mg tablets
46638	124021	Paracetamol 500mg / Ibuprofen 200mg tablets
29704	59460020	Paxofen 200mg Tablet (M A Steinhard Ltd)
11952	65700020	Phenylbutazone 100mg gastro-resistant tablets
29010	90907020	Phenylbutazone 100mg tablets
27723	65701020	Phenylbutazone 200mg tablets
28695	79273020	Piroflam 10mg Capsule (Opus Pharmaceuticals Ltd)
19320	79274020	Piroflam 20mg Capsule (Opus Pharmaceuticals Ltd)
20663	!5677102	PIROXICAM
44703	50314020	Piroxicam 10mg Capsule (Berk Pharmaceuticals Ltd)
141	65858020	Piroxicam 10mg capsules
41622	50328020	Piroxicam 10mg capsules (A A H Pharmaceuticals Ltd)
43541	50320020	Piroxicam 10mg capsules (Actavis UK Ltd)
41624	54331020	Piroxicam 10mg capsules (IVAX Pharmaceuticals UK Ltd)
2463	68349020	Piroxicam 10mg dispersible tablets
77185	69505020	Piroxicam 20mg Capsule (Ashbourne Pharmaceuticals Ltd)
21123	50315020	Piroxicam 20mg Capsule (Berk Pharmaceuticals Ltd)
1755	65859020	Piroxicam 20mg capsules
41621	50329020	Piroxicam 20mg capsules (A A H Pharmaceuticals Ltd)
29465	50321020	Piroxicam 20mg capsules (Actavis UK Ltd)
74659	54593020	Piroxicam 20mg capsules (Approved Prescription Services Ltd)
41623	54332020	Piroxicam 20mg capsules (IVAX Pharmaceuticals UK Ltd)
37750	60589020	Piroxicam 20mg capsules (Mylan)
3710	68350020	Piroxicam 20mg dispersible tablets
67608	56350020	Piroxicam 20mg dispersible tablets (A A H Pharmaceuticals Ltd)
31777	60595020	Piroxicam 20mg dispersible tablets (Mylan)
4965	65860020	Piroxicam 20mg orodispersible tablets sugar free
11495	50157020	Piroxicam betadex 20mg tablets
20699	!5677103	PIROXICAM DISPERSIBLE
20742	!5677104	PIROXICAM DISPERSIBLE
21864	56769020	Pirozip 10 capsules (Ashbourne Pharmaceuticals Ltd)
21846	56770020	Pirozip 20 capsules (Ashbourne Pharmaceuticals Ltd)
126	51186020	Ponstan 250mg capsules (Chemidex Pharma Ltd)
1246	51187020	Ponstan 250mg Dispersible tablet (Chemidex Pharma Ltd)
14541	53445020	Ponstan 50mg/5ml paediatric Liquid (Chemidex Pharma Ltd)
296	51182020	Ponstan Forte 500mg tablets (Chemidex Pharma Ltd)
21843	57369020	Pranoxen continus 375mg Tablet (Napp Pharmaceuticals Ltd)
21816	57370020	Pranoxen continus 500mg Tablet (Napp Pharmaceuticals Ltd)
9474	81066020	Preservex 100mg tablets (Almirall Ltd)

19575	63156020	Proflex 200mg Tablet (Novartis Consumer Health UK Ltd)
30811	63157020	Proflex 300mg Modified-release capsule (Novartis Consumer Health UK Ltd)
17754	51288020	Progesic 200mg Tablet (Eli Lilly and Company Ltd)
33111	56630020	Prosaid 250mg Tablet (BHR Pharmaceuticals Ltd)
23323	56631020	Prosaid 500mg Tablet (BHR Pharmaceuticals Ltd)
28519	75925020	Pseudoephedrine 30mg with ibuprofen 200mg tablet
27438	75926020	Pseudoephedrine 45mg with ibuprofen 300mg modified-release capsule
32366	82148020	Relcofen 200mg Tablet (Actavis UK Ltd)
32365	82149020	Relcofen 400mg tablets (Actavis UK Ltd)
16473	60242020	Relifex 500mg dispersible tablets (Meda Pharmaceuticals Ltd)
2235	67974020	Relifex 500mg tablets (Meda Pharmaceuticals Ltd)
10295	67975020	Relifex 500mg/5ml oral suspension (Meda Pharmaceuticals Ltd)
25750	57373020	Rheuflex 250mg Tablet (Goldshield Pharmaceuticals Ltd)
28816	57374020	Rheuflex 500mg Tablet (Goldshield Pharmaceuticals Ltd)
26351	83497020	Rheumatac Retard 75 tablets (AMCo)
10296	4453007	RHEUMOX 100 MG CAP
3739	53354020	Rheumox 300mg capsules (Mercury Pharma Group Ltd)
7688	51376020	Rheumox 600mg tablets (Mercury Pharma Group Ltd)
25790	68882020	Rhumalgan 25mg Tablet (Lagap)
30806	68883020	Rhumalgan 50mg Tablet (Lagap)
21610	80211020	Rhumalgan CR 100 tablets (Sandoz Ltd)
17029	80210020	Rhumalgan CR 75 tablets (Sandoz Ltd)
56898	15708021	Rhumalgan SR 75mg capsules (Actavis UK Ltd)
47501	59021	Rhumalgan SR 75mg capsules (Almus Pharmaceuticals Ltd)
17030	88558020	Rhumalgan SR 75mg capsules (Sandoz Ltd)
56078	61021	Rhumalgan XL 100mg capsules (Almus Pharmaceuticals Ltd)
26631	88560020	Rhumalgan XL 100mg capsules (Sandoz Ltd)
21419	89574020	Seractil 300mg tablets (Thornton & Ross Ltd)
21421	89582020	Seractil 400mg tablets (Thornton & Ross Ltd)
24201	6994007	SLOFENAC 100 MG TAB
24236	83889020	Slofenac 100mg Modified-release tablet (Sterwin Medicines)
21620	6993007	SLOFENAC 75 MG TAB
19382	83888020	Slofenac 75mg SR tablets (Sterwin Medicines)
18922	87111020	Solpadeine Headache soluble tablets (Omega Pharma Ltd)
10196	87109020	Solpadeine Headache tablets (GlaxoSmithKline Consumer Healthcare)
39461	90563020	Solpadeine Migraine Ibuprofen & Codeine tablets (Omega Pharma Ltd)
10178	89794020	Solpadeine Plus capsules (Omega Pharma Ltd)
10226	89796020	Solpadeine Plus tablets (Omega Pharma Ltd)

25330	82982020	Solpaflex tablets (GlaxoSmithKline Consumer Healthcare)
67117	61302021	Stirlescent 250mg effervescent tablets (Stirling Anglian Pharmaceuticals Ltd)
44892	99013020	Sudafed sinus pressure & pain Tablet (McNeil Products Ltd)
20907	82037020	Sudafed Sinus Pressure & Pain tablets (McNeil Products Ltd)
3897	66832020	Sulindac 100mg tablets
5482	66833020	Sulindac 200mg tablets
27916	!6853201	SURGAM
387	51745020	Surgam 200mg tablets (Sanofi)
25643	51747020	Surgam 300mg Sachets (Sanofi)
1778	51746020	Surgam 300mg Tablet (Sanofi)
14776	77350020	Surgam 300mg tablets (Sanofi)
2257	67923020	Surgam SA 300mg capsules (Sanofi)
3817	57767020	Synflex 275mg tablets (Roche Products Ltd)
24682	69417020	Tenoxicam 20mg effervescent tablets
47816	66615020	Tenoxicam 20mg Tablet (Sovereign Medical Ltd)
3974	69415020	Tenoxicam 20mg tablets
27013	86742020	Tiloket 200mg Modified-release capsule (Tillomed Laboratories Ltd)
31916	86741020	Tiloket CR 100mg capsules (Tillomed Laboratories Ltd)
31429	86902020	Timpron 250mg Gastro-resistant tablet (Berk Pharmaceuticals Ltd)
26242	74810020	Timpron 250mg Tablet (Berk Pharmaceuticals Ltd)
26231	74811020	Timpron 500mg Gastro-resistant tablet (Berk Pharmaceuticals Ltd)
26216	74809020	Timpron 500mg Tablet (Berk Pharmaceuticals Ltd)
18640	51959020	Tolectin 200mg Capsule (Cilag Pharmaceuticals Ltd)
10711	51960020	Tolectin 400mg Capsule (Cilag Pharmaceuticals Ltd)
15159	83568020	Tolfenamic acid 200mg Capsule
7222	83569020	Tolfenamic acid 200mg tablets
22410	5629007	TOLMETIN 200 MG TAB
26404	67137020	Tolmetin 200mg Capsule
20016	67138020	Tolmetin 400mg Capsule
3336	69061020	Toradol 10mg tablets (Roche Products Ltd)
29037	81085020	Valdic 100 Retard tablets (Fannin UK Ltd)
30849	81084020	Valdic 75 Retard tablets (Fannin UK Ltd)
28390	73897020	Valenac ec 25mg Gastro-resistant tablet (Shire Pharmaceuticals Ltd)
25283	73898020	Valenac ec 50mg Gastro-resistant tablet (Shire Pharmaceuticals Ltd)
57943	98801020	Valket 200 Retard capsules (Tillomed Laboratories Ltd)
24020	57154020	Valrox 250mg Tablet (Shire Pharmaceuticals Ltd)
24007	57155020	Valrox 500mg Tablet (Shire Pharmaceuticals Ltd)

44986	99220020	Vimovo 500mg/20mg modified-release tablets (AstraZeneca UK Ltd)
21444	74266020	Volraman 25mg gastro-resistant tablets (LPC Medical (UK) Ltd)
15201	74267020	Volraman 50mg gastro-resistant tablets (LPC Medical (UK) Ltd)
11168	80723020	Volsaid Retard 100 tablets (Chiesi Ltd)
4506	80722020	Volsaid Retard 75 tablets (Chiesi Ltd)
497	83709020	Voltarol 25mg gastro-resistant tablets (Novartis Pharmaceuticals UK Ltd)
1139	53288020	Voltarol 25mg Tablet (Novartis Pharmaceuticals UK Ltd)
50058	8086020	Voltarol 50mg dispersible tablets (DE Pharmaceuticals)
49059	8090020	Voltarol 50mg dispersible tablets (Lexon (UK) Ltd)
589	72769020	Voltarol 50mg dispersible tablets (Novartis Pharmaceuticals UK Ltd)
4631	83710020	Voltarol 50mg gastro-resistant tablets (Novartis Pharmaceuticals UK Ltd)
1446	53289020	Voltarol 50mg Tablet (Novartis Pharmaceuticals UK Ltd)
4625	83774020	Voltarol 75mg SR tablets (Novartis Pharmaceuticals UK Ltd)
44112	98747020	Voltarol Joint Pain 12.5mg tablets (Novartis Consumer Health UK Ltd)
39722	95573020	Voltarol Pain-eze 12.5mg tablets (Novartis Consumer Health UK Ltd)
47820	164021	Voltarol Pain-eze Extra Strength 25mg tablets (Novartis Consumer Health UK Ltd)
5401	82738020	Voltarol Rapid 25mg tablets (Novartis Pharmaceuticals UK Ltd)
53345	8375020	Voltarol Rapid 50mg tablets (Lexon (UK) Ltd)
51099	8377020	Voltarol Rapid 50mg tablets (Mawdsley-Brooks & Company Ltd)
5085	82739020	Voltarol Rapid 50mg tablets (Novartis Pharmaceuticals UK Ltd)
70145	8376020	Voltarol Rapid 50mg tablets (Stephar (U.K.) Ltd)
27901	!7713301	VOLTAROL RETARD
2386	53298020	Voltarol Retard 100mg tablets (Novartis Pharmaceuticals UK Ltd)
1766	53294020	Voltarol sr 75mg Modified-release tablet (Novartis Pharmaceuticals UK Ltd)

S3: Code list for upper GI bleed outcome.

ICD-10 code	Description
K25.0	Gastric ulcer Acute with haemorrhage
K25.2	Gastric ulcer Acute with both haemorrhage and perforation
K25.4	Gastric ulcer Chronic or unspecified with haemorrhage
K25.6	Gastric ulcer Chronic or unspecified with both haemorrhage and perforation
K26.0	Duodenal ulcer Acute with haemorrhage
K26.2	Duodenal ulcer Acute with both haemorrhage and perforation
K26.4	Duodenal ulcer Chronic or unspecified with haemorrhage
K26.6	Duodenal ulcer Chronic or unspecified with both haemorrhage and perforation
K27.0	Peptic ulcer, site unspecified Acute with haemorrhage
K27.2	Peptic ulcer, site unspecified Acute with both haemorrhage and perforation
K27.4	Peptic ulcer, site unspecified Chronic or unspecified with haemorrhage
K27.6	Peptic ulcer, site unspecified Chronic or unspecified with both haemorrhage and perforation
K28.0	Gastrojejunal ulcer Acute with haemorrhage
K28.2	Gastrojejunal ulcer Acute with both haemorrhage and perforation
K28.4	Gastrojejunal ulcer Chronic or unspecified with haemorrhage
K28.6	Gastrojejunal ulcer Chronic or unspecified with both haemorrhage and perforation
K29.0	Acute haemorrhagic gastritis
K92.0	Haematemesis
K92.1	Melaena
K92.2	Gastrointestinal haemorrhage, unspecified

S4: Characteristics of NSAID and COX-2 inhibitor users in unmatched, matched samples using Prescription-based exposure-set definition. The unmatched NSAID user group was generated by sampling one NSAID comparator prescription from each exposure set before matching.

Abbreviations: ASD, Absolute standardised differences; BMI, body mass index; CKD, chronic kidney disease; GERD, gastroesophageal reflux disease; H2RA, h2 receptor antagonists; IBD, inflammatory bowel disease; IMD, index of multiple deprivation; OCS, oral corticosteroids; PPI, proton pump inhibitor; SD, standard deviation; SSRI, Selective serotonin reuptake inhibitors; UGIB, upper gastrointestinal bleeding

	Unmatched	Matched		
	NSAID	NSAID	COX-2i	ASD
N	37385	37385	37385	
Age (Mean (SD))	65.20 (12.71)	69.06 (11.92)	69.46 (11.98)	0.033
Male	15383 (41.1)	12002 (32.1)	12040 (32.2)	0.002
Urban	5769 (15.4)	6393 (17.1)	6479 (17.3)	0.006
IMD				0.024
1	4285 (11.5)	4190 (11.2)	4306 (11.5)	
2	4263 (11.4)	4209 (11.3)	4109 (11.0)	
3	4176 (11.2)	4312 (11.5)	4205 (11.2)	
4	4118 (11.0)	4203 (11.2)	4202 (11.2)	
5	4108 (11.0)	4083 (10.9)	4262 (11.4)	
6	3900 (10.4)	3895 (10.4)	3846 (10.3)	
7	3508 (9.4)	3619 (9.7)	3555 (9.5)	
8	3463 (9.3)	3445 (9.2)	3486 (9.3)	
9	2733 (7.3)	2694 (7.2)	2615 (7.0)	
10	2831 (7.6)	2735 (7.3)	2799 (7.5)	
Hospital Admissions				0.022
0	32653 (87.3)	31986 (85.6)	31728 (84.9)	
1	3590 (9.6)	4064 (10.9)	4317 (11.5)	
2	752 (2.0)	880 (2.4)	887 (2.4)	
>2	390 (1.0)	455 (1.2)	453 (1.2)	
Alcohol Consumption				0.009
High	627 (1.7)	529 (1.4)	526 (1.4)	
Low	18204 (48.7)	19967 (53.4)	19804 (53.0)	
Missing	18554 (49.6)	16889 (45.2)	17055 (45.6)	
Smoking Status				0.027
Current	5575 (14.9)	5306 (14.2)	5138 (13.7)	
Ex	6797 (18.2)	7805 (20.9)	7575 (20.3)	
Non-smoker	18408 (49.2)	18815 (50.3)	18937 (50.7)	

Missing	6605 (17.7)	5459 (14.6)	5735 (15.3)	
Body Mass Index				0.013
<18.5	257 (0.7)	314 (0.8)	290 (0.8)	
18.5-25	10052 (26.9)	10041 (26.9)	10061 (26.9)	
25-30	12549 (33.6)	12601 (33.7)	12437 (33.3)	
30+	6833 (18.3)	7098 (19.0)	7163 (19.2)	
Missing	7694 (20.6)	7331 (19.6)	7434 (19.9)	
Comorbidities				
IBD	304 (0.8)	387 (1.0)	379 (1.0)	0.002
Heart Failure	1299 (3.5)	1745 (4.7)	1886 (5.0)	0.018
Hypertension	12270 (32.8)	14727 (39.4)	14828 (39.7)	0.006
GI Cancer	386 (1.0)	414 (1.1)	437 (1.2)	0.006
CKD	141 (0.4)	198 (0.5)	184 (0.5)	0.005
Diabetes	2931 (7.8)	3160 (8.5)	3247 (8.7)	0.008
Coronary Angioplasty	180 (0.5)	261 (0.7)	255 (0.7)	0.002
Coagulopathy	174 (0.5)	213 (0.6)	240 (0.6)	0.009
Previous UGIB	772 (2.1)	1190 (3.2)	1282 (3.4)	0.014
GERD	1280 (3.4)	2046 (5.5)	2165 (5.8)	0.014
Medications/Therapies				
Statin	3922 (10.5)	5200 (13.9)	5120 (13.7)	0.006
PPI/H2RA	3667 (9.8)	6781 (18.1)	7159 (19.1)	0.026
SSRI	1804 (4.8)	1949 (5.2)	2130 (5.7)	0.021
Anticoagulant	267 (0.7)	518 (1.4)	555 (1.5)	0.008
Antiplatetes	5837 (15.6)	7479 (20.0)	7718 (20.6)	0.016
OCS	974 (2.6)	1493 (4.0)	1575 (4.2)	0.011
Other Respiratory	3186 (8.5)	4184 (11.2)	4234 (11.3)	0.004
Calendar Year				0.185
2000	6789 (18.2)	3512 (9.4)	3003 (8.0)	
2001	8758 (23.4)	6253 (16.7)	6231 (16.7)	
2002	7953 (21.3)	7622 (20.4)	9212 (24.6)	
2003	7301 (19.5)	9010 (24.1)	10492 (28.1)	
2004	6584 (17.6)	10988 (29.4)	8447 (22.6)	

S5: Characteristics of NSAID and COX-2 inhibitor users in unmatched, matched samples using Time exposure-set definition with a 30-day caliper. The unmatched NSAID user group was generated by sampling one NSAID comparator prescription from each exposure set before matching.

Abbreviations: ASD, Absolute standardised differences; BMI, body mass index; CKD, chronic kidney disease; GERD, gastroesophageal reflux disease; H2RA, h2 receptor antagonists; IBD, inflammatory bowel disease; IMD, index of multiple deprivation; OCS, oral corticosteroids; PPI, proton pump inhibitor; SD, standard deviation; SSRI, Selective serotonin reuptake inhibitors; UGIB, upper gastrointestinal bleeding

	Unmatched	Matched		
	NSAID	NSAID	COX-2i	ASD
N	37549	37549	37549	
Age (Mean (SD))	65.43 (12.86)	68.66 (11.98)	69.56 (11.96)	0.075
Male	15193 (40.5)	12454 (33.2)	12071 (32.1)	0.022
Urban	6008 (16.0)	6311 (16.8)	6541 (17.4)	0.016
IMD				0.024
1	4098 (10.9)	4244 (11.3)	4326 (11.5)	
2	4149 (11.0)	4157 (11.1)	4127 (11.0)	
3	4356 (11.6)	4297 (11.4)	4200 (11.2)	
4	4226 (11.3)	4257 (11.3)	4221 (11.2)	
5	4031 (10.7)	4162 (11.1)	4300 (11.5)	
6	3932 (10.5)	3937 (10.5)	3841 (10.2)	
7	3575 (9.5)	3472 (9.2)	3582 (9.5)	
8	3620 (9.6)	3446 (9.2)	3502 (9.3)	
9	2736 (7.3)	2762 (7.4)	2636 (7.0)	
10	2826 (7.5)	2815 (7.5)	2814 (7.5)	
Hospital Admissions				0.039
0	32607 (86.8)	32302 (86.0)	31811 (84.7)	
1	3697 (9.8)	3916 (10.4)	4364 (11.6)	
2	839 (2.2)	887 (2.4)	909 (2.4)	
>2	406 (1.1)	444 (1.2)	465 (1.2)	
Alcohol Consumption				0.006
High	551 (1.5)	554 (1.5)	530 (1.4)	
Low	18561 (49.4)	19892 (53.0)	19929 (53.1)	
Missing	18437 (49.1)	17103 (45.5)	17090 (45.5)	
Smoking Status				0.037
Current	5460 (14.5)	5316 (14.2)	5164 (13.8)	
Ex	6848 (18.2)	7927 (21.1)	7611 (20.3)	
Non-smoker	18497 (49.3)	18980 (50.5)	19018 (50.6)	
Missing	6744 (18.0)	5326 (14.2)	5756 (15.3)	

Body Mass Index				0.018
<18.5	238 (0.6)	284 (0.8)	294 (0.8)	
18.5-25	10110 (26.9)	10110 (26.9)	10062 (26.8)	
25-30	12507 (33.3)	12586 (33.5)	12477 (33.2)	
30+	6838 (18.2)	7329 (19.5)	7223 (19.2)	
Missing	7856 (20.9)	7240 (19.3)	7493 (20.0)	
Comorbidities				
IBD	294 (0.8)	371 (1.0)	386 (1.0)	0.004
Heart Failure	1369 (3.6)	1688 (4.5)	1916 (5.1)	0.028
Hypertension	12356 (32.9)	14686 (39.1)	14997 (39.9)	0.017
GI Cancer	398 (1.1)	394 (1.0)	446 (1.2)	0.013
CKD	145 (0.4)	173 (0.5)	185 (0.5)	0.005
Diabetes	2953 (7.9)	3168 (8.4)	3295 (8.8)	0.012
Coronary Angioplasty	188 (0.5)	282 (0.8)	260 (0.7)	0.007
Coagulopathy	176 (0.5)	208 (0.6)	239 (0.6)	0.011
Previous UGIB	778 (2.1)	1125 (3.0)	1306 (3.5)	0.027
GERD	1291 (3.4)	2035 (5.4)	2166 (5.8)	0.015
Medications/Therapies				
Statin	3986 (10.6)	5282 (14.1)	5198 (13.8)	0.006
PPI/H2RA	4012 (10.7)	6445 (17.2)	7394 (19.7)	0.065
SSRI	1894 (5.0)	2003 (5.3)	2141 (5.7)	0.016
Anticoagulant	351 (0.9)	489 (1.3)	564 (1.5)	0.017
Antiplatetes	5970 (15.9)	7327 (19.5)	7795 (20.8)	0.031
OCS	1049 (2.8)	1434 (3.8)	1620 (4.3)	0.025
Other Respiratory	3359 (8.9)	3952 (10.5)	4293 (11.4)	0.029
Calendar Year				0.208
2000	7800 (20.8)	3132 (8.3)	3003 (8.0)	
2001	8842 (23.5)	5828 (15.5)	6250 (16.6)	
2002	7961 (21.2)	7540 (20.1)	9306 (24.8)	
2003	6883 (18.3)	9295 (24.8)	10510 (28.0)	
2004	6063 (16.1)	11754 (31.3)	8480 (22.6)	

S6: Characteristics of NSAID and COX-2 inhibitor users in unmatched, matched samples using Hybrid exposure-set definition with a 15-day caliper. The unmatched NSAID user group was generated by sampling one NSAID comparator prescription from each exposure set before matching.

Abbreviations: ASD, Absolute standardised differences; BMI, body mass index; CKD, chronic kidney disease; GERD, gastroesophageal reflux disease; H2RA, h2 receptor antagonists; IBD, inflammatory bowel disease; IMD, index of multiple deprivation; OCS, oral corticosteroids; PPI, proton pump inhibitor; SD, standard deviation; SSRI, Selective serotonin reuptake inhibitors; UGIB, upper gastrointestinal bleeding

	Unmatched	Matched		
	NSAID	NSAID	COX-2i	ASD
N	32789	32789	32789	
Age (Mean (SD))	64.65 (12.91)	69.70 (11.81)	69.60 (11.92)	0.008
Male	13320 (40.6)	10494 (32.0)	10539 (32.1)	0.003
Urban	5125 (15.6)	5655 (17.2)	5735 (17.5)	0.006
IMD				0.025
1	3727 (11.4)	3661 (11.2)	3758 (11.5)	
2	3635 (11.1)	3630 (11.1)	3586 (10.9)	
3	3862 (11.8)	3797 (11.6)	3658 (11.2)	
4	3724 (11.4)	3706 (11.3)	3679 (11.2)	
5	3628 (11.1)	3648 (11.1)	3771 (11.5)	
6	3221 (9.8)	3457 (10.5)	3339 (10.2)	
7	3143 (9.6)	3074 (9.4)	3170 (9.7)	
8	3080 (9.4)	3042 (9.3)	3084 (9.4)	
9	2353 (7.2)	2355 (7.2)	2326 (7.1)	
10	2416 (7.4)	2419 (7.4)	2418 (7.4)	
Hospital Admissions				0.031
0	28758 (87.7)	28173 (85.9)	27867 (85.0)	
1	3068 (9.4)	3450 (10.5)	3766 (11.5)	
2	652 (2.0)	774 (2.4)	778 (2.4)	
>2	311 (0.9)	392 (1.2)	378 (1.2)	
Alcohol Consumption				0.007
High	509 (1.6)	473 (1.4)	448 (1.4)	
Low	16207 (49.4)	17254 (52.6)	17280 (52.7)	
Missing	16073 (49.0)	15062 (45.9)	15061 (45.9)	
Smoking Status				0.006
Current	5129 (15.6)	4491 (13.7)	4476 (13.7)	
Ex	6290 (19.2)	6429 (19.6)	6449 (19.7)	
Non-smoker	15821 (48.3)	16666 (50.8)	16601 (50.6)	

Missing	5549 (16.9)	5203 (15.9)	5263 (16.1)	
Body Mass Index				0.014
<18.5	220 (0.7)	261 (0.8)	257 (0.8)	
18.5-25	9119 (27.8)	8687 (26.5)	8851 (27.0)	
25-30	10907 (33.3)	11056 (33.7)	10865 (33.1)	
30+	5846 (17.8)	6189 (18.9)	6175 (18.8)	
Missing	6697 (20.4)	6596 (20.1)	6641 (20.3)	
Comorbidities				
IBD	268 (0.8)	315 (1.0)	332 (1.0)	0.005
Heart Failure	1057 (3.2)	1632 (5.0)	1674 (5.1)	0.006
Hypertension	10540 (32.1)	13101 (40.0)	12983 (39.6)	0.007
GI Cancer	354 (1.1)	329 (1.0)	382 (1.2)	0.016
CKD	128 (0.4)	160 (0.5)	159 (0.5)	<0.001
Diabetes	2469 (7.5)	2788 (8.5)	2861 (8.7)	0.008
Coronary Angioplasty	182 (0.6)	201 (0.6)	216 (0.7)	0.006
Coagulopathy	168 (0.5)	177 (0.5)	201 (0.6)	0.01
Previous UGIB	686 (2.1)	1033 (3.2)	1093 (3.3)	0.01
GERD	1264 (3.9)	1707 (5.2)	1823 (5.6)	0.016
Medications/Therapies				
Statin	3899 (11.9)	4304 (13.1)	4389 (13.4)	0.008
PPI/H2RA	3019 (9.2)	5894 (18.0)	6003 (18.3)	0.009
SSRI	1505 (4.6)	1784 (5.4)	1853 (5.7)	0.009
Anticoagulant	317 (1.0)	467 (1.4)	511 (1.6)	0.011
Antiplatetes	5136 (15.7)	6639 (20.2)	6702 (20.4)	0.005
OCS	802 (2.4)	1342 (4.1)	1345 (4.1)	<0.001
Other Respiratory	2794 (8.5)	3714 (11.3)	3723 (11.4)	0.001
Calendar Year				<0.001
2000	2710 (8.3)	2989 (9.1)	2989 (9.1)	
2001	5399 (16.5)	6148 (18.8)	6148 (18.8)	
2002	7714 (23.5)	8817 (26.9)	8817 (26.9)	
2003	8698 (26.5)	8616 (26.3)	8616 (26.3)	
2004	8268 (25.2)	6219 (19.0)	6219 (19.0)	

S7: Characteristics of NSAID and COX-2 inhibitor users in unmatched, matched samples using Time exposure-set definition with a 15-day caliper. The unmatched NSAID user group was generated by sampling one NSAID comparator prescription from each exposure set before matching.

Abbreviations: ASD, Absolute standardised differences; BMI, body mass index; CKD, chronic kidney disease; GERD, gastroesophageal reflux disease; H2RA, h2 receptor antagonists; IBD, inflammatory bowel disease; IMD, index of multiple deprivation; OCS, oral corticosteroids; PPI, proton pump inhibitor; SD, standard deviation; SSRI, Selective serotonin reuptake inhibitors; UGIB, upper gastrointestinal bleeding

	Unmatched	Matched		
	NSAID	NSAID	COX-2i	ASD
N	36224	36224	36224	
Age (Mean (SD))	65.27 (12.91)	68.89 (11.98)	69.65 (11.96)	0.064
Male	14725 (40.6)	12090 (33.4)	11592 (32.0)	0.029
Urban	5776 (15.9)	6105 (16.9)	6346 (17.5)	0.018
IMD				0.021
1	4339 (12.0)	4053 (11.2)	4169 (11.5)	
2	3960 (10.9)	3979 (11.0)	3971 (11.0)	
3	4147 (11.4)	4232 (11.7)	4060 (11.2)	
4	4134 (11.4)	4098 (11.3)	4068 (11.2)	
5	3883 (10.7)	4023 (11.1)	4137 (11.4)	
6	3713 (10.3)	3721 (10.3)	3713 (10.3)	
7	3469 (9.6)	3436 (9.5)	3461 (9.6)	
8	3312 (9.1)	3392 (9.4)	3391 (9.4)	
9	2588 (7.1)	2603 (7.2)	2542 (7.0)	
10	2679 (7.4)	2687 (7.4)	2712 (7.5)	
Hospital Admissions				0.031
0	31613 (87.3)	31029 (85.7)	30700 (84.8)	
1	3517 (9.7)	3856 (10.6)	4206 (11.6)	
2	722 (2.0)	904 (2.5)	878 (2.4)	
>2	372 (1.0)	435 (1.2)	440 (1.2)	
Alcohol Consumption				0.007
High	581 (1.6)	498 (1.4)	504 (1.4)	
Low	17722 (48.9)	19103 (52.7)	19215 (53.0)	
Missing	17921 (49.5)	16623 (45.9)	16505 (45.6)	
Smoking Status				0.025
Current	5318 (14.7)	5106 (14.1)	4947 (13.7)	
Ex	6435 (17.8)	7480 (20.6)	7259 (20.0)	
Non-smoker	17893 (49.4)	18219 (50.3)	18367 (50.7)	

Missing	6578 (18.2)	5419 (15.0)	5651 (15.6)	
Body Mass Index				0.018
<18.5	266 (0.7)	265 (0.7)	287 (0.8)	
18.5-25	10033 (27.7)	9562 (26.4)	9718 (26.8)	
25-30	11833 (32.7)	12295 (33.9)	12039 (33.2)	
30+	6568 (18.1)	6950 (19.2)	6924 (19.1)	
Missing	7524 (20.8)	7152 (19.7)	7256 (20.0)	
Comorbidities				
IBD	273 (0.8)	345 (1.0)	373 (1.0)	0.008
Heart Failure	1349 (3.7)	1677 (4.6)	1859 (5.1)	0.023
Hypertension	11797 (32.6)	14200 (39.2)	14486 (40.0)	0.016
GI Cancer	420 (1.2)	388 (1.1)	427 (1.2)	0.01
CKD	132 (0.4)	162 (0.4)	175 (0.5)	0.005
Diabetes	2672 (7.4)	3039 (8.4)	3174 (8.8)	0.013
Coronary Angioplasty	202 (0.6)	226 (0.6)	257 (0.7)	0.011
Coagulopathy	143 (0.4)	210 (0.6)	234 (0.6)	0.008
Previous UGIB	735 (2.0)	1091 (3.0)	1277 (3.5)	0.029
GERD	1211 (3.3)	1928 (5.3)	2106 (5.8)	0.021
Medications/Therapies				
Statin	3762 (10.4)	5015 (13.8)	4977 (13.7)	0.003
PPI/H2RA	3542 (9.8)	6497 (17.9)	7187 (19.8)	0.049
SSRI	1739 (4.8)	1971 (5.4)	2083 (5.8)	0.013
Anticoagulant	303 (0.8)	486 (1.3)	557 (1.5)	0.016
Antiplatetes	5647 (15.6)	7133 (19.7)	7545 (20.8)	0.028
OCS	935 (2.6)	1439 (4.0)	1582 (4.4)	0.02
Other Respiratory	2956 (8.2)	3912 (10.8)	4163 (11.5)	0.022
Calendar Year				0.231
2000	7560 (20.9)	3257 (9.0)	3003 (8.3)	
2001	8881 (24.5)	5872 (16.2)	6250 (17.3)	
2002	7493 (20.7)	7331 (20.2)	9306 (25.7)	
2003	6519 (18.0)	8902 (24.6)	10163 (28.1)	
2004	5771 (15.9)	10862 (30.0)	7502 (20.7)	

Figures

Figure S1: Forest plot of estimated hazard ratios for association between COX2-i versus NSAID use and UGIB from sensitivity analyses varying the time caliper for the time-based and hybrid exposure set definitions from 30-days to 15-days. Three estimands are presented: i) Effect of incident new-use or switching (Overall), ii) Effect of prevalent new-use (i.e. switching), and iii) Effect of incident new-use.

Analysis	Hazard Ratio (95% CI)
Time-based (15-days): Overall	1.07 (0.79 – 1.45)
Prevalent New-users	1.82 (1.04 – 3.21)
Incident New-users	0.81 (0.56 – 1.17)
Hybrid-based (15-days): Overall	1.07 (0.77 – 1.48)
Prevalent New-users	1.24 (0.64 – 2.38)
Incident New-users	1.00 (0.68 – 1.47)

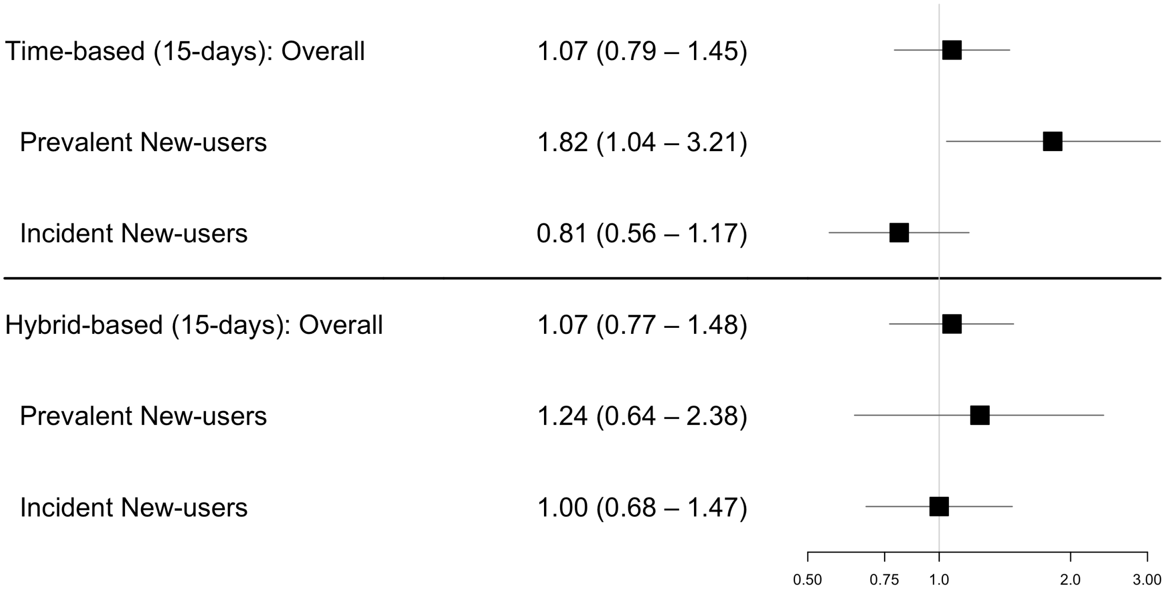

Supplement: Supplementary file 1 — Data S1: pds70339‐sup‐0001‐Supinfo.pdf. [file PDS-35-e70339-s001.pdf]
